# Microcosm experiment combined with process-based modeling reveals differential response and adaptation of aquatic primary producers to warming and agricultural run-off

**DOI:** 10.3389/fpls.2023.1120441

**Published:** 2023-06-19

**Authors:** Gregorio A. López Moreira Mazacotte, Bastian H. Polst, Elisabeth M. Gross, Mechthild Schmitt-Jansen, Franz Hölker, Sabine Hilt

**Affiliations:** ^1^ Department of Community and Ecosystem Ecology, Leibniz Institute of Freshwater Ecology and Inland Fisheries (IGB), Berlin, Germany; ^2^ Department of Bioanalytical Ecotoxicology, Helmholtz Centre for Environmental Research-UFZ, Leipzig, Germany; ^3^ Laboratoire Interdisciplinaire des Environnements Continentaux (LIEC) UMR 7360 CNRS, Université de Lorraine, Metz, France; ^4^ LTSER Zone Atelier Bassin de la Moselle, Metz, France

**Keywords:** climate change, regime shifts, alternative stable states, stress-induced tolerance, mathematical modeling, multiple stressors, shallow lakes, phytoplankton succession

## Abstract

Fertilizers, pesticides and global warming are threatening freshwater aquatic ecosystems. Most of these are shallow ponds or slow-flowing streams or ditches dominated by submerged macrophytes, periphyton or phytoplankton. Regime shifts between the dominance of these primary producers can occur along a gradient of nutrient loading, possibly triggered by specific disturbances influencing their competitive interactions. However, phytoplankton dominance is less desirable due to lower biodiversity and poorer ecosystem function and services. In this study, we combined a microcosm experiment with a process-based model to test three hypotheses: 1) agricultural run-off (ARO), consisting of nitrate and a mixture of organic pesticides and copper, differentially affects primary producers and enhances the risk of regime shifts, 2) warming increases the risk of an ARO-induced regime shift to phytoplankton dominance and 3) custom-tailored process-based models support mechanistic understanding of experimental results through scenario comparison. Experimentally exposing primary producers to a gradient of nitrate and pesticides at 22°C and 26°C supported the first two hypotheses. ARO had direct negative effects on macrophytes, while phytoplankton gained from warming and indirect effects of ARO like a reduction in the competitive pressure exerted by other groups. We used the process-based model to test eight different scenarios. The best qualitative fit between modeled and observed responses was reached only when taking community adaptation and organism acclimation into account. Our results highlight the importance of considering such processes when attempting to predict the effects of multiple stressors on natural ecosystems.

## 1 Introduction

The overall share of land used for crops and pastures is increasing worldwide (Winkler et al., 2021). However, global cropland per capita is steadily decreasing as the world's population continues to grow (FAO, 2021), leading to a rapid increase in the global use of pesticides and fertilizers (Sharma et al., 2019). In addition, climate change is increasing the average temperature of most inland water bodies (O’Reilly et al., 2015). Together, these multiple stressors may severely affect aquatic ecosystems in agricultural areas. Many of these agroecosystems are shallow ponds or slow-flowing streams and are characterized by the alternative dominance of submerged macrophytes, periphyton or phytoplankton, competing for light and nutrients as major resources (Vasconcelos et al., 2016). Shifts from submerged macrophyte dominance to the less desirable phytoplankton dominance have been reported with increased nutrient loading (Sayer et al., 2010). Warming is expected to lead to more severe phytoplankton dominance (modeling study by Mooij et al., 2007) and to increase periphyton growth (Mahdy et al., 2015; Kazanjian et al., 2018), resulting in an overall weakening of the resilience of macrophyte-dominated systems to nutrient loading impacts (Meerhoff et al., 2022). Additional stressors such as pesticides, however, may antagonistically interact with temperature and nutrient loading, modulating the competition for resources between aquatic primary producers (Polst et al., 2022). Understanding the overall response of these ecosystems to multiple stressors is therefore challenging (Jackson et al., 2016) but crucial to predict future changes in their functioning (Hilt et al., 2017) and services (Janssen et al., 2021).

A first experiment investigating responses of the different primary producers typical for shallow aquatic systems to multiple stressors revealed a direct positive effect of co-occurring nitrate and pesticides on phytoplankton, with no significant effect of warming (Allen et al., 2021). A subsequent study, involving also primary consumers, showed that pesticides and nitrate may interact synergistically to reduce macrophyte dominance, and that pesticides and warming may have especially strong but opposite effects on specific macrophyte species (Vijayaraj et al., 2022). Climate warming has even been shown to lower critical thresholds for shifts in dominance between aquatic primary producers (Polst et al., 2022). These differences may derive from the differential sensitivities to toxicants of different groups and species of primary producers (Fairchild et al., 1998; Giddings et al., 2013), some of which may be more tolerant or have the ability to acclimate or adapt to them at different temperature-dependent rates (Chalifour and Juneau, 2011; Larras et al., 2013). Additional trophic levels, the structure of the ecosystem and the trophic status of the water body may also play a role (Wendt-Rasch et al., 2004), further complicating the analysis of direct and indirect effects of multiple stressors that may lead to complex responses of shallow aquatic ecosystems.

Reductionist factorial laboratory approaches are useful to address related research questions. However, time and budget constraints, and the invasive nature of macrophyte sampling often limit both the number of testable stressor combinations and the frequency of sampling that would be needed to understand community dynamics. Combining experiments with process-based models may be a helpful tool to reveal variable trajectories, to test for critical threshold values, and to disentangle indirect stressor effects. Such models, however, need to be developed based on the specific research questions and the experimental set-up, i.e., custom-tailored to avoid running into equifinality issues (López Moreira et al., 2021). The empirical results, in turn, allow for subsequent model refinement, calibration and validation (e.g., Kim et al., 2021), an iterative process that builds upon every new insight and gained expert knowledge (Jakeman et al., 2006).

In this study, we combined a microcosm experiment on multiple stressor effects in shallow aquatic ecosystems with simulations we ran with a custom-tailored process-based model. We developed this model to investigate the response of the different primary producer groups (submerged macrophytes, periphyton and phytoplankton) to the combined effects of nitrate and pesticides typically found in agricultural run-off (ARO) with or without effects of climate warming. We hypothesized: 1) that ARO differentially affects primary producer groups increasing the likelihood of phytoplankton dominance in shallow aquatic ecosystems; 2) that warming facilitates this process and 3) that custom-tailored process-based models can support mechanistic understanding of experimental results through scenario comparison. First, we developed a simple process-based mathematical model based on the known effects of herbicides, fertilizers and warming on phytoplankton, periphyton and macrophytes. To assess model predictions, we carried out a microcosm experiment testing the response of three submerged macrophyte species (*Myriophyllum spicatum*, *Potamogeton perfoliatus* and *Elodea nuttallii*) and mixtures of phytoplankton and periphyton typically occurring in European freshwaters. We exposed these systems to a gradient of an experimental ARO cocktail, an artificial mix of organic pesticides (an herbicide, an insecticide and a fungicide), copper-(II) sulfate (CuSO_4_) and potassium nitrate (KNO_3_) for 19 days. The ARO mix was applied in a two-factor factorial design (dose-response set-up) at two different temperatures (ambient: 22°C, warming: 26°C). Experimental results led us to refine the process-based model and test eight scenarios of differential herbicide sensitivities among primary producer groups, temperature dependence of the response, and development of tolerance to the herbicide for three cases (sets) of phytoplankton community composition. Comparing *in silico* simulations with experimental results allowed us to select the best fit and improved mechanistic understanding of differential sensitivities of phytoplankton, periphyton and submerged macrophytes to combined ARO and warming.

## 2 Materials and methods

### 2.1 Process-based model to simulate the microcosms

#### 2.1.1 Governing and supplementary equations

To simulate the combined effects of ARO and warming on the different groups of aquatic primary producers, we developed a process-based mathematical model, which we implemented in MATLAB (R2020a). The model comprises a series of ordinary differential equations (ODEs) similar to those proposed in previous works (Jäger et al., 2010; Vasconcelos et al., 2016). These equations describe the following state variables: the live carbon (C) biomass of phytoplankton (as volume concentration, eq. 1, specific to each phytoplankton group), periphyton, hereafter understood as the growth on the vertical inner surface of the microcosm vases (as areal density, eq. 2), epiphyton, i.e., periphyton growing on macrophyte surfaces (as areal density, eq. 3) and macrophytes (eq. 4). Because exudates and lysates resulting from growth and senescence, respectively, are known to support microbial communities (Kieft et al., 2021), the model also includes governing equations for the C content of the cellular exudates of all primary producer groups (as volume concentration, eqs. 5-8) and for their dead C biomass (consistent units, eqs. 9-12). These processes were included because the presence of pesticides in ARO and an increased temperature may have important direct effects on decomposers (e.g., heterotrophic bacteria and fungi) and, consequently, on the rates of nutrient recycling within the microcosms (Nielsen, 2006). Additional governing equations describe the areal density of dead phytoplankton cells accumulating in the sediments (eq. 13, specific to each phytoplankton group), and the volume concentration of dissolved inorganic phosphorus (eq. 14). The resulting system of ODEs is:


$$\{\begin{array}{ll} \frac{dC_{live,phyto}}{dt}=\left(p_{phyto}-l_{phyto}-\frac{w_{live,phyto}}{H_{V}}\right)C_{live,phyto} & \left(1\right)\\ \frac{dC_{live,peri}}{dt}=\left(p_{peri}-l_{peri}\right)C_{live,peri} & \left(2\right)\\ \frac{dC_{live,epi}}{dt}=\left(p_{epi}-l_{epi}\right)C_{live,epi} & \left(3\right)\\ \frac{dC_{live,macro}}{dt}=\left(p_{macro}-l_{macro}\right)C_{live,macro} & \left(4\right)\\ \frac{dC_{exu,phyto}}{dt}=l_{exu,phyto}C_{live,phyto}-b_{exu,phyto}C_{exu,phyto} & \left(5\right)\\ \frac{dC_{exu,peri}}{dt}=l_{exu,peri}C_{live,peri}\frac{A_{peri}}{V}-b_{exu,peri}C_{exu,peri} & \left(6\right)\\ \frac{dC_{exu,epi}}{dt}=l_{exu,epi}C_{live,epi}\frac{A_{epi}}{V}-b_{exu,epi}C_{exu,epi} & \left(7\right)\\ \frac{dC_{exu,macro}}{dt}=l_{exu,macro}C_{live,macro}\frac{1}{V}-b_{exu,macro}C_{exu,macro} & \left(8\right)\\ \frac{dC_{dead,phyto}}{dt}=l_{d,phyto}C_{live,phyto}-\left(\frac{w_{dead,phyto}}{H_{V}}+b_{dead,phyto}\right)C_{dead,phyto} & \left(9\right)\\ \frac{dC_{dead,peri}}{dt}=l_{d,peri}C_{live,peri}-b_{dead,peri}C_{dead,peri} & \left(10\right)\\ \frac{dC_{dead,epi}}{dt}=l_{d,epi}C_{live,epi}-b_{dead,epi}C_{dead,epi} & \left(11\right)\\ \frac{dC_{dead,macro}}{dt}=l_{d,macro}C_{live,macro}-b_{dead,macro}C_{dead,macro} & \left(12\right)\\ \frac{dC_{dead,sed,phyto}}{dt}=w_{live,phyto}C_{live,phyto}+w_{dead,phyto}C_{dead,phyto}\\ -b_{dead,phyto}C_{dead,sed,phyto} & \left(13\right)\\ \frac{dP_{d}}{dt}=\;S_{phyto}+S_{peri}+S_{epi}+S_{macro} & \left(14\right) \end{array}$$

where the parameters $p_{i=phyto,peri,epi,macro}$ and $l_{i=phyto,peri,epi,macro}$ (eqs. 1-4) are variable total unit gain and unit loss rates of C biomass of each group; $w_{live,phyto}$ (eqs. 1,13) and $w_{dead,phyto}$ (eqs. 9,13) are constant sinking velocities of live and dead phytoplankton cells, respectively; $H_{V}$ is the constant height of water in the microcosm of constant water volume V ; the $l_{exu,i=phyto,peri,epi,macro}\;$ (eqs. 5-8) are the variable unit background exudation rates of each group; the $b_{exu,i=phyto,peri,epi,macro}$ (eqs. 5-8) are the variable unit biodegradation rates of exudates; the $b_{dead,i=phyto,peri,epi,macro}$ (eqs. 9-13) are the variable unit biodegradation rates of dead biomass; $A_{peri}$ is the constant area of the microcosm surface where wall periphyton can grow; and $A_{epi}$ the variable area of macrophyte surfaces where epiphyton can grow, formulated as a function of macrophyte biomass. The time coordinate and all state variables (governing eqs. 1-14) are listed and described in **Table 1**. Primary auxiliary variables, i.e., those appearing in the governing equations of the model, are listed and described in **Table 2**. The formulations of all secondary auxiliary variables appearing in the equations of primary auxiliary variables are presented as part of the supplementary information (SI), as well as the chosen values of all model parameters (**Tables S1**–**S6**) and the initial values of all state variables (**Table S7**).

**Table 1:** Description, units of measurement of the time coordinate and state variables, and reference to the governing equations.

Variable	Description	Units	Formulation
Time coordinate			
*t*	Time coordinate, with origin at the start of the simulated period and positive direction forward	s	–
State variables			
*C_live,phyto_*	Volume concentration of live carbon biomass of phytoplankton	mg C m^-3^	Eq. 1
*C_live,peri_*	Areal density of live carbon biomass of periphyton	mg C m^-2^	Eq. 2
*C_live,epi_*	Areal density of live carbon biomass of epiphyton	mg C m^-2^	Eq. 3
*C_live,macro_*	Live carbon biomass of macrophytes	mg C	Eq. 4
*C_exu,phyto_*	Volume concentration of carbon in phytoplankton exudates	mg C m^-3^	Eq. 5
*C_exu,peri_*	Volume concentration of carbon in periphyton exudates	mg C m^-3^	Eq. 6
*C_exu,epi_*	Volume concentration of carbon in epiphyton exudates	mg C m^-3^	Eq. 7
*C_exu,macro_*	Volume concentration of carbon in macrophyte exudates	mg C m^-3^	Eq. 8
*C_dead,phyto_*	Volume concentration of dead carbon biomass of phytoplankton	mg C m^-3^	Eq. 9
*C_dead,peri_*	Areal density of dead carbon biomass of periphyton	mg C m^-2^	Eq. 10
*C_dead,epi_*	Areal density of dead carbon biomass of periphyton	mg C m^-2^	Eq. 11
*C_dead,macro_*	Dead carbon biomass of macrophytes	mg C	Eq. 12
*C_dead,sed,phyto_*	Areal density of dead carbon biomass of phytoplankton that is stored in the sediments	mg C m^-2^	Eq. 13
*P_d_*	Volume concentration of dissolved inorganic phosphorus	mg P m^-3^	Eq. 14

**Table 2:** Description, units of measurement of primary auxiliary variables (appearing in the governing equations), and reference to their formulation.

Variable	Description	Units	Formulation
*p_phyto_*	Fractional growth rate of carbon biomass of phytoplankton groups	s^-1^	Eq. S15
*p_peri_*	Fractional growth rate of carbon biomass of periphyton	s^-1^	Eq. S19
*p_epi_*	Fractional growth rate of carbon biomass of epiphyton	s^-1^	Eq. S20
*A_epi_*	Area of macrophyte surfaces where epiphyton can grow	m^2^	Eq. S24
*p_macro_*	Fractional growth rate of carbon biomass of macrophytes	s^-1^	Eq. S25
*l_i_*_=_ *_phyto,peri,epi,macro_*	Fractional loss rate of carbon biomass of primary producers	s^-1^	Eq. S28
*l_d,i_*_=_ *_phyto,peri,epi,macro_*	Fractional death rate of carbon biomass of primary producers	s^-1^	Eq. S29
*l_exu,i_*_=_ *_phyto,peri,epi,macro_*	Fractional exudation rate of carbon biomass of primary producers	s^-1^	Eq. S30
*b_dead,i=phyto,peri,epi,macro_*	Fractional biodegradation rate of dead carbon biomass of primary producers	s^-1^	Eq. S34
*b_exu,i=phyto,peri,epi,macro_*	Fractional biodegradation rate of cellular exudates of primary producers	s^-1^	Eq. S35
*S_phyto_*	Source/sink of phosphorus, phytoplankton	mg P m^-3^	Eq. S36
*S_peri_*	Source/sink of phosphorus, periphyton	mg P m^-3^	Eq. S37
*S_epi_*	Source/sink of phosphorus, epiphyton	mg P m^-3^	Eq. S38
*S_macro_*	Source/sink of phosphorus, macrophytes	mg P m^-3^	Eq. S39

Note that, in the model, periphyton and epiphyton are treated as separate primary producer groups. While periphyton grows on the vertical inner glass surface of the microcosm, which is of constant area, epiphyton grows on macrophyte surfaces that change over time as macrophytes develop. We implemented a space limitation factor in the auxiliary equation for the growth rates of periphyton and epiphyton (eqs. S19 to S22). Additionally, the growth rates of all primary producers are also dependent on nutrient and light availability, and are affected by temperature. On this note, in general, all modelled processes are accelerated under warmer conditions, as described in the SI. The effect of the herbicide was modeled based on log-logistic dose-response curves that are common in toxicokinetic studies (e.g., Copin and Chèvre, 2015). These curves were made specific to each group of primary producers, as described in the SI (**Figure S1**).

### 2.2 Microcosm experiment

#### 2.2.1 Microcosm setup

To mimic shallow aquatic ecosystems, we used microcosms based on the OECD Guideline 239 *Water-Sediment Myriophyllum Spicatum Toxicity Test* (Allen et al., 2021; Polst et al., 2022; Vijayaraj et al., 2022). The microcosms consisted of glass vases (diameter: 25 cm, height: 40 cm, manufacturer: Sandra Rich, Germany) containing a glass bowl (diameter: 14 cm, height: 8 cm, manufacturer: Sandra Rich, Germany) filled with 20% Kaolin (Imerys, France), 5% peat (<1 mm), 1% nettle powder, 74% quartz sand (grain size fraction<0.2 mm, manufacturer: Schicker Mineral GmbH, Germany) and a 2 cm layer of quartz sand (<0.2 mm) on top to prevent resuspension of the sediment.

Three submerged macrophyte species that are common in temperate eutrophic freshwater ecosystems (Hilt et al., 2018) were collected from nearby eutrophic water bodies: *Myriophyllum spicatum* and *Elodea nuttallii* from Lake Müggelsee (Germany) and *Potamogeton perfoliatus* from River Spree (Germany). Two apical shoots of 8 cm (*M. spicatum*, *E. nuttallii*) or 10 cm (*P. perfoliatus*) were planted into the sediments of each microcosm.

All microcosms were filled with 8 L of Volvic^®^ mineral water (Danone Waters Deutschland GmbH, Germany) to ensure homogeneity and low initial nutrient concentrations (NO_3_-: 7.3 mg L^−1^). After one week of initial adjustment of the system, the following species of photoautotrophic microorganisms were added, that are typical of shallow freshwater ecosystems: four planktonic species – including two cyanobacteria (*Chroococcus minutus* and *Anabaena* sp. PCC7210) and two green algae (*Scenedesmus obliquus* and *Desmodesmus subspicatus*) – and four benthic species – including a diatom (*Nitzschia palea*), two filamentous green algae (*Uronema* sp. and *Oedogonium* sp.) and a cyanobacterium (*Komvophorum* sp.). The species were selected and grown in Volvic^®^ water prior to inoculation, and then combined for an inoculum with a total biovolume of 1.25×10^9^ µm^3^. Three plastic strips (30×2.5 cm) were fixed in the sediment bowl and attached to the inner glass vase, providing a surface for periphyton growth (150 cm^2^ each). An aeration system was constructed to ensure mixing of the water within each microcosm using air pumps.

The microcosms were placed inside two climate chambers, each containing a reference temperature sensor in one of the microcosms. Heating and cooling of the microcosms happened *via* air temperature changes. A 16h:8h light:dark cycle was applied using luminescent light with a mean of 77.2 ± 9.9 µmol m^-2^ s^-1^ measured at the water surface.

#### 2.2.2 ARO composition

The artificial mixture of pesticides representing a characteristic agricultural run-off (ARO) was selected as in our previous studies (e.g., Allen et al., 2021), including three organic pesticides – terbuthylazine (selective chloro-s-triazine herbicide, PSII inhibitor, CAS number 5915-41-3), pirimicarb (fast-acting selective carbamate insecticide, AChE inhibitor, CAS number 23103-98-2) and tebuconazole (triazole fungicide, demethylation inhibitor, CAS number 107534-96-3) – copper (inorganic pesticide, as CuSO_4_) and nitrate (fertilizer, as KNO_3_). The initial concentrations of pesticides were based on dose-response assays and subsequent EC20 calculations (as described in Allen et al., 2021). An initial concentration of 9 mg L^-1^ NO_3_-N was selected as representative for nitrogen concentrations in small lakes in agricultural catchments during spring and summer (James et al., 2005; Xu et al., 2014). For the application in the microcosms, the organic pesticides were diluted in dimethyl sulfoxide (DMSO, Sigma-Aldrich, 67-68-5, final concentration in the microcosm 0.1%). CuSO_4_ and KNO_3_ were diluted in ultrapure water.

#### 2.2.3 Two-factor factorial dose-response design

The experiment was conducted in two climate chambers (22°C, 26°C) and in each, we followed a dose-response setup for ARO with one control and five concentration levels (ARO 1x, 2x, 4x, 8x, 16x; enrichment factor of 2). The ARO mixture and concentrations were chosen based on former work by Allen et al. (2021). The ARO concentration used in their study was set as intermediate treatment concentration in our experiment (ARO 4x) with two higher and two lower concentrations for the gradient. Each treatment had five to seven replicates.

#### 2.2.4 Time schedule

The bowls filled with sediment were prepared and pre-wetted with Volvic^®^ mineral water and stored in dark conditions at 22°C for two days before macrophytes were planted and microcosms were filled with Volvic^®^ mineral water. Afterwards, macrophytes were given two weeks to adjust to the conditions. The inoculum of microscopic primary producers and plastic strips were added one week before the start of the experiment. At the start of the experiment, treatments were applied by adding the ARO mix to the microcosms and increasing the temperature in one of the climate chambers to 26°C. Hereafter, nutrients in the form of a KNO_3_ and KH_2_PO_4_ mixture as in Allen et al. (2021) were added twice a week, simulating repeated nutrient loading to compensate for a fast nutrient uptake by wall periphyton, and thus ensure sufficient nutrient availability to sustain further primary producer growth. On days 16, 17 and 18, we sampled periphyton and phytoplankton, and took water samples for chemical analysis (day 18). On day 19, to end the experiment, the lights were turned off and sampling of the macrophytes took place until day 23 (**Figure S2**).

#### 2.2.5 Sampling methods

##### 2.2.5.1 Biomass sampling of primary producers

Periphyton was brushed off from the plastic strips using a toothbrush and suspended in Volvic^®^ water. This periphyton-suspension was then filtered with pre-weighted glass fiber filters (0.7 µm), dried at 60°C and weighted to determine the periphyton dry weight. To determine phytoplankton biomass, water samples were filtered using pre-weighted glass fiber filters (0.7 µm), dried at 60°C and weighted. Macrophytes were removed from their microcosm for the final sampling and the aboveground part of each macrophyte specimen was separately packed in paper bags, dried at 60°C for two days and then weighted to determine the dry weight of each stem.

##### 2.2.5.2 Water sampling for pesticide concentrations

Two hours and 18 days after application of the treatments, 4 mL-water subsamples of three microcosms per ARO treatment were taken and frozen at −20°C. Later, the samples were filtered (0.2 µm) and analyzed for their concentration of the three pesticides used in the ARO mixture. Measurement of the pesticides was conducted using a UltiMate3000 HPLC System combined with an LTQ-OrbiTrap XL (Thermo Scientific, USA). These samples were then analyzed with an UltiMate3000 HPLC System (column: Phenomenex, Art.-No. 00B-4462-Y0) and an attached LTQ Orbitrap XL (Thermo Scientific) operated in positive ionization mode.

#### 2.2.6 Statistical analyses

Non-parametric statistical tests were used to test for differences between treatments related to the ARO application or the temperature. These analyses were conducted using the software R (R Core Team, 2020; v4.0.0). For each of the three primary producer groups differences in biomass were tested *via* the Kruskal-Wallis test followed by Dunn's *post hoc* test without further correction due to the low number of treatments.

Further, biomass of each primary producer group was extrapolated to the whole biomass per microcosm, including periphyton growing on the inner walls of the microcosm. Based on this total microcosm primary producer biomass, the effect sizes (as Hedges’ *g*) were calculated using the *esc* package (Lüdecke, 2019; v0.5.1). Using the mean biomass of each primary producer group as well as the total biomass of all primary producer groups together, pie charts were created for simplified presentation of the share of each primary producer group within each treatment.

### 2.3 Model refinement and assessment against experimental results

The information we progressively gained over the course of the experiment and after experimental results became available allowed for further refinement of the model to account for some processes like the decay of toxicant concentrations over time (revealed by water sampling results), or to better represent processes like nutrient uptake by wall periphyton, nutrient recycling due to biodegradation, and trait-based phytoplankton community adaptation by splitting the phytoplankton compartment into two groups. Group *α* was conceived as a fast-growing phytoplankton group that was highly sensitive to the herbicide (i.e., low EC50 value), whereas group *β* was made up of slow-growing phytoplankton that were resistant to the herbicide (i.e., a much higher EC50 value than group *α*).

Moreover, the log-logistic dose-response curves were made variable in time to account for the potential development of tolerance (adaptation) to the herbicide and/or acclimation of the organisms to better cope with the combined stressors. We assumed these adaptation and acclimation processes to be directly linked to the generation time of the organisms, i.e., faster for microscopic primary producers. This was achieved by implementing dynamic parameters for the dose-response curves, as described in the SI. The potential of the refined model to simulate community adaptation, tolerance development and organism acclimation led us to design and test the model under different scenarios of herbicide sensitivity, temperature dependence of primary producer response and adaptation and/or acclimation.

#### 2.3.1 Scenarios of differential sensitivities, temperature dependence, adaptation and/or acclimation

We designed eight different scenarios to test our hypotheses and to mechanistically understand experimental results. These scenarios related to: a) whether microscopic primary producers (phytoplankton group *α*, periphyton and epiphyton) were equally, more or less sensitive to the herbicide than macrophytes; b) whether these sensitivities were influenced by temperature or not; and c) whether sensitivities decreased over time or not as a result of the development of tolerance to the herbicide by adaptation or acclimation of microscopic primary producers to environmental conditions (**Table 3**).

**Table 3:** Scenarios of differential sensitivities, temperature dependence, adaptation and/or acclimation.

Scenario	Herbicide sensitivity by group	Effect of temperature on herbicide sensitivity
A1	Fast-growing phytoplankton (group *α*), wall periphyton, epiphyton and macrophytes are equally sensitive to the herbicide. Slow-growing phytoplankton (group *β*) are less sensitive to the herbicide than all other groups. Sensitivities do not decrease over time for any group (no adaptation or acclimation).	NO
A2		Sensitivities are higher at the lower temperature (22°C)
B1	Macrophytes are more sensitive to the herbicide than all microscopic primary producers. Slow-growing phytoplankton (group *β*) are less sensitive to the herbicide than all other groups. Sensitivities do not decrease over time (no adaptation or acclimation).	NO
B2		Sensitivities are higher at the lower temperature (22°C)
C1	Fast-growing phytoplankton (group *α*), wall periphyton and epiphyton are more sensitive to the herbicide than macrophytes. Slow-growing phytoplankton (group *β*) are less sensitive to the herbicide than all other groups. Sensitivities do not decrease over time (no adaptation or acclimation).	NO
C2		Sensitivities are higher at the lower temperature (22°C)
D1	Initially, fast-growing phytoplankton (group *α*), periphyton and epiphyton are more sensitive to the herbicide than macrophytes. These groups become less sensitive to the herbicide over time to become less sensitive than macrophytes by the end of the exposure. From the beginning of the exposure, low-growing phytoplankton (group *β*) are less sensitive to the herbicide than all other groups and become increasingly tolerant to it over time.	NO
D2		Sensitivities are higher at the lower temperature (22°C)

In scenarios A1 and A2, all primary producer groups were equally sensitive to the herbicide except for phytoplankton group *β* (conceived as slow-growing and resistant to the herbicide). In scenarios B1 and B2, macrophytes were more sensitive to the herbicide than all other primary producer groups. In scenarios C1 and C2, microscopic primary producers were more sensitive to the herbicide than macrophytes, with the exception of phytoplankton group *β*, which, again, was more resistant than all other groups.

In all aforementioned scenarios (A1-2, B1-2 and C1-2), we assumed that no development of tolerance to the herbicide by acclimation or adaptation to environmental conditions (Lips et al., 2022) occurred over the course of the exposure, i.e., sensitivities do not decrease over time. In scenarios D1 and D2, however, we account for this by implementing decreasing herbicide sensitivities over the course of the exposure for all microscopic primary producers. In these two scenarios, phytoplankton group *α*, periphyton and epiphyton start off being more sensitive to the herbicide than macrophytes, but end up becoming less sensitive than macrophytes by the end of the simulated period as a result of tolerance development. Here too, phytoplankton group *β* starts off being less sensitive than all other groups, and becomes increasingly tolerant over time.

In all scenarios indexed with “1”, we assumed that temperature had no effect at all on herbicide sensitivities, whereas in all scenarios indexed with “2”, sensitivities to the herbicide were assumed to be higher at the lower temperature (22°C) than at the higher temperature (26°C), following empirical evidence by Tasmin et al., 2013. Moreover, this is consistent with the fact that metabolic processes are in general accelerated at higher temperatures (Clarke and Fraser, 2004; Pedrosa Gomes and Juneau, 2017).

To further account for possible community adaptation and succession of phytoplankton to the applied stressors related to an increasing dominance of the more tolerant species (Blanck, 2002; Tlili et al., 2016; Lips et al., 2022), we ran three sets of simulations under all eight scenarios. Set 1: a single phytoplankton group that is fast-growing and highly sensitive to the herbicide, hereafter referred to as group *α*; Set 2: a single phytoplankton group that is slow-growing but much less sensitive to the herbicide, hereafter referred to as group *β*; Set 3: a mixed community of both phytoplankton groups *α* and *β*, as previously described.

#### 2.3.2 Assessment of model simulation results

We assessed the goodness of fit between model results and the observations from the microcosm experiment following a pattern-oriented model validation approach that took the ARO treatment as predictor of final biomass values for each of the following three primary producer groups: phytoplankton, periphyton (wall growth in the model, periphyton strip growth in the experiment) and macrophytes. The resulting patterns were compared qualitatively in terms of the similarity between simulated and observed response curves and quantitatively on the basis of the correlation coefficients calculated for all scenarios between model results and empirical observations.

## 3 Results

### 3.1 Microcosm experiment

#### 3.1.1 Biomass of different primary producer groups

**Figure 1:**
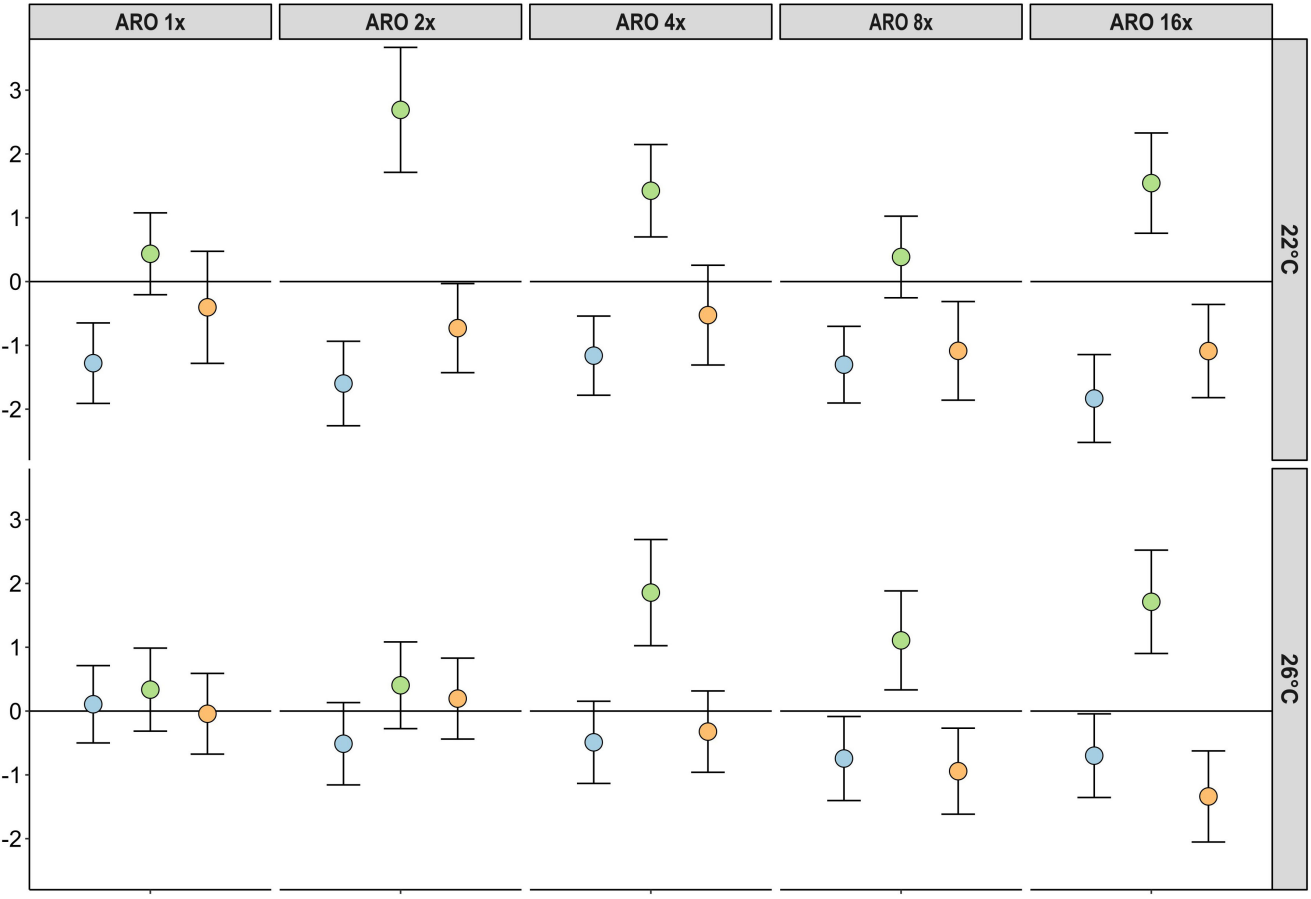
Effect sizes (Hedges’ *g*) and its standard deviation for the response of different primary producer groups (blue = macrophytes, green = phytoplankton, orange = periphyton) and ARO treatment levels at 22°C (top row) and 26°C (bottom row).

**Figure 2:**
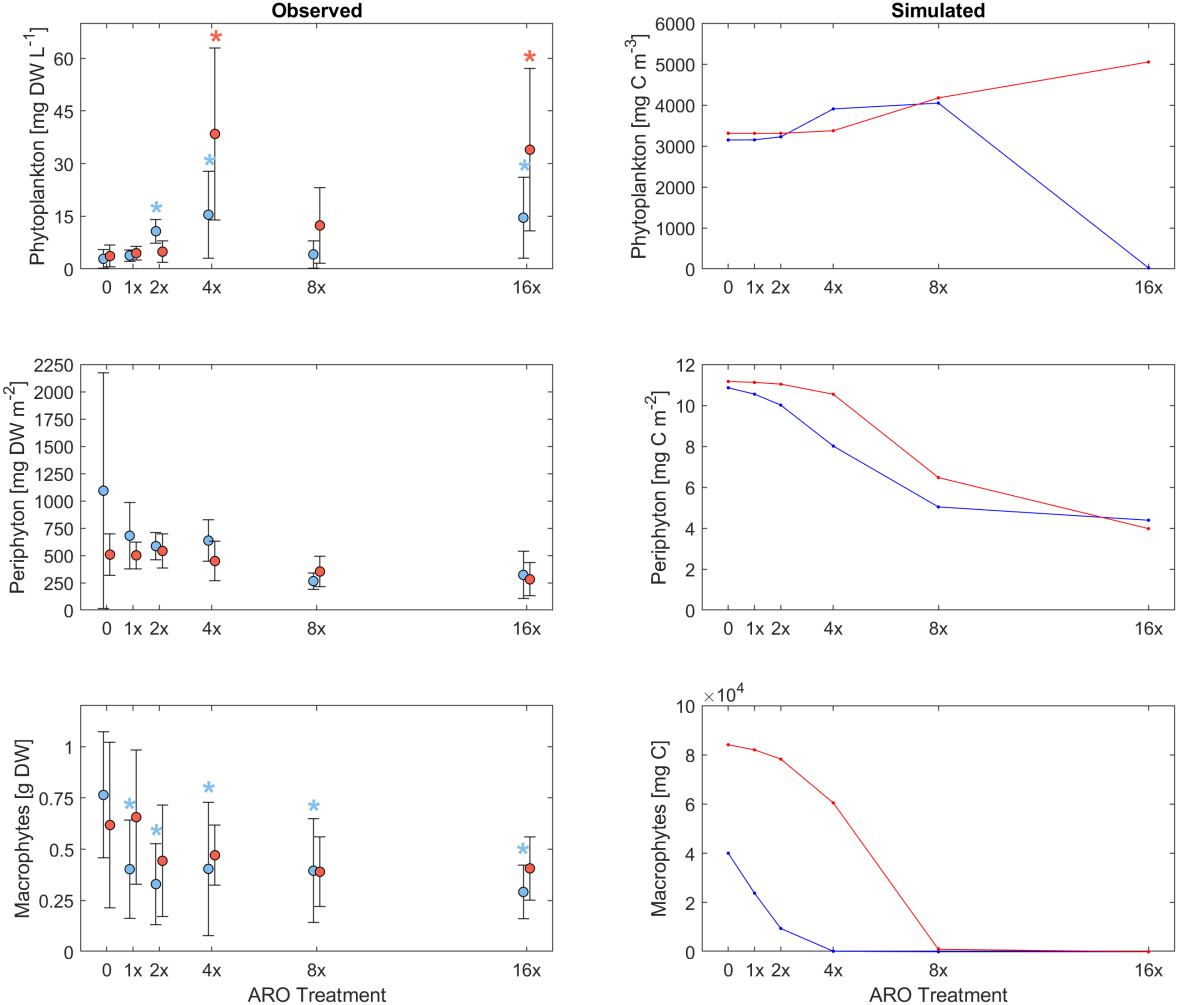
Response of different primary producer groups to multiple stressors by increasing concentrations of pesticides and nutrients in agricultural runoff (ARO) and warming (+4°C) observed in experimental microcosms (left) and simulated (right) for a mixed phytoplankton community (set 3) under the most plausible scenario (D2) for warm (26°C, red) and ambient (22°C, blue) temperature treatments. Asterisks indicate significant differences with respect to the control.

At 22°C, all ARO treatments showed significantly lower macrophyte biomass than the controls (p-value < 0.05), but did not significantly differ among the five ARO treatments. At 26°C, macrophyte biomass across all ARO concentrations was not significantly different in relation to the controls or the respective ARO concentrations at 22°C (**Figures 1**, **2** and **Table 4**). At 22°C, phytoplankton biomass showed a significant increase compared with the controls at the second tested ARO concentration level (ARO 2x) and higher (apart from ARO 8x). At 26°C, phytoplankton biomass was significantly higher at ARO 4x and ARO 16x. No significant differences between the respective ARO concentrations at the two temperatures were detected (**Figures 1**, **2**). Periphyton biomass was not significantly affected by ARO neither at 22°C nor at 26°C, likely due to the high variation among replicates in the controls (**Figure 2**). Effect sizes for periphyton showed negative effects at the two highest tested ARO levels for both temperatures (**Figure 1**).

**Table 4:** Nominal concentrations of all ARO constituents and measured concentrations 2 hours after its application (n=3, ARO 8: n=1,< d.l. = below detection limit). Concentrations in µg L^-1^.

Treatment	Terbuthylazine	Pirimicarb	Tebuconazole	Copper	NO_3_-N
Control	0 (< d.l.)	0 (< d.l.)	0 (< d.l.)	0 (< d.l.)	0 (< d.l.)
ARO 1x	0.75 | 0.64 ± 0.01	3.75 | 3.82 ± 0.22	22.5 | 21.11 ± 1.21	10.5	2250
ARO 2x	1.5 | 1.28 ± 0.06	7.5 | 7.24 ± 0.14	45 | 38.07 ± 4.00	21	4500
ARO 4x	3 | 2.74 ± 0.06	15 | 15.56 ± 0.62	90 | 83.46 ± 6.56	42	9000
ARO 8x	6 | 5.70 ± 0.00	30 | 31.07 ± 0.00	180 | 171.80 ± 0.00	84	18000
ARO 16x	12 | 11.6±+0.59	60 | 63.64 ± 3.02	360 | 323.03 ± 23.06	168	36000

**Figure 3:**
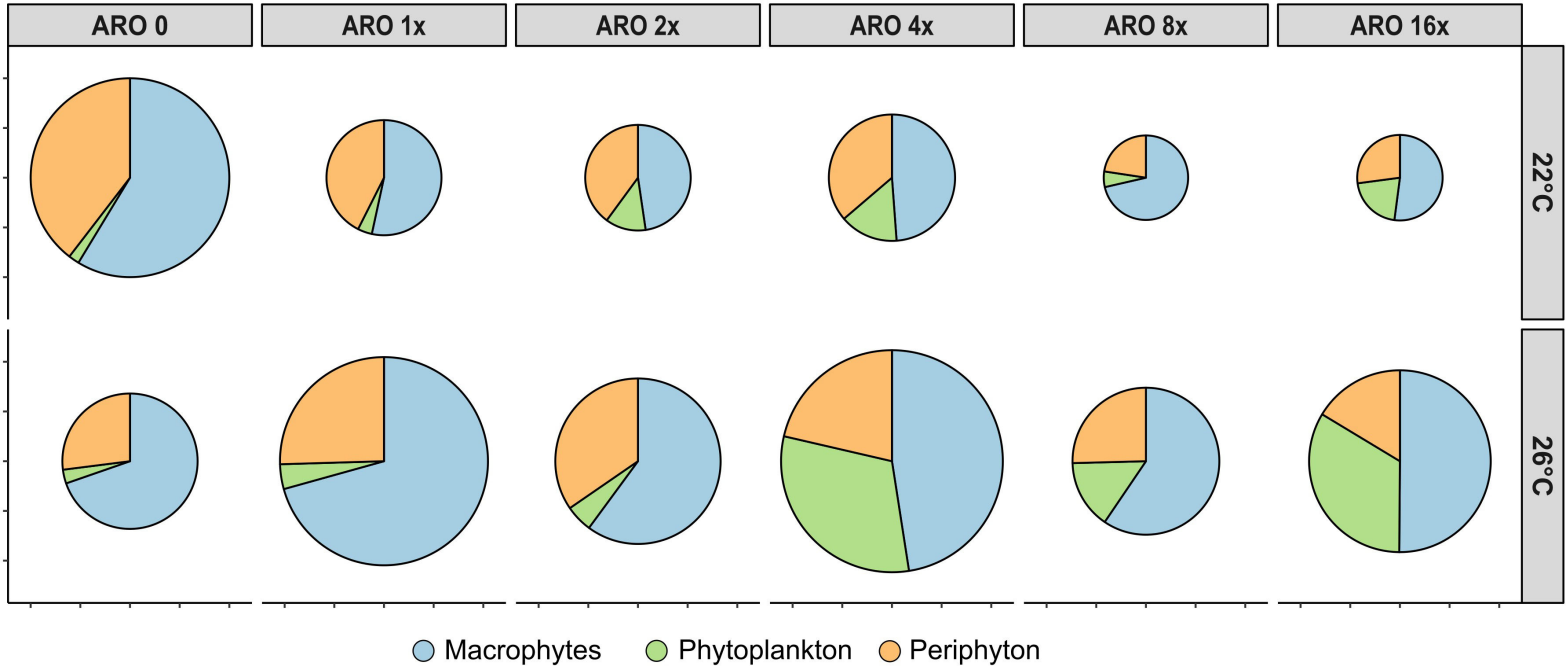
Biomass proportions of the three primary producer groups upscaled to whole microcosm biomass. Size of the pies indicate relative total biomass in comparison with the controls (ARO 0) at 22°C.

The average accumulated microcosm biomass for all primary producers decreased in the ARO treatments compared with the control at 22°C. At 26°C, the accumulated microcosm biomass of the ARO treatments was higher than their control at 26°C and the respective ARO treatments at 22°C. (**Figure 3**). In the control treatment, macrophytes dominated with periphyton having the second highest share. The share of both macrophytes and periphyton decreased with ARO treatments due to increasing phytoplankton abundance, with the lowest share of macrophytes on the accumulated microcosm biomass at ARO 4x (**Figure 3**). The share of phytoplankton increased at 26°C.

#### 3.1.2 Change in pesticide concentrations

After two hours of exposure, the pesticide concentrations were approximately 10% lower than the nominal concentration (**Table 4**). One microcosm showed higher concentrations than planned (and wrong ratios between the three pesticides) and was, thereafter, excluded from further analysis. At the end of the experiment, between 55-90% of the initial pesticide concentrations were still detected (**Table S8**). No temperature-related differences in the final pesticide concentrations were found.

### 3.2 Process-based microcosm model

**Figure 4:**
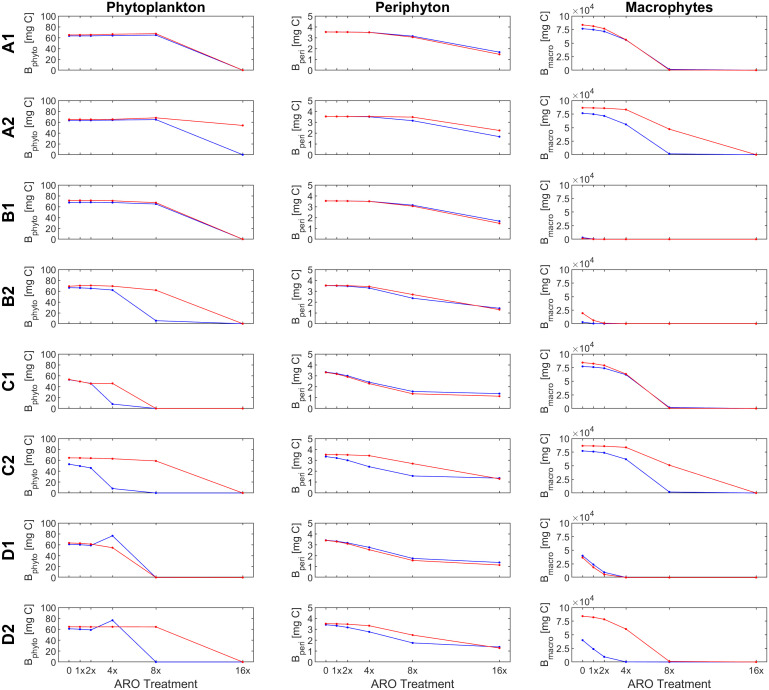
Response of primary producer groups under the eight scenarios (A1-D2) for the case of a single, fast-growing phytoplankton group that is highly sensitive to the herbicide (simulation set 1, group *α* only) under ambient (22°C, blue) and warm (26°C, red) temperature conditions.

**Figure 5:**
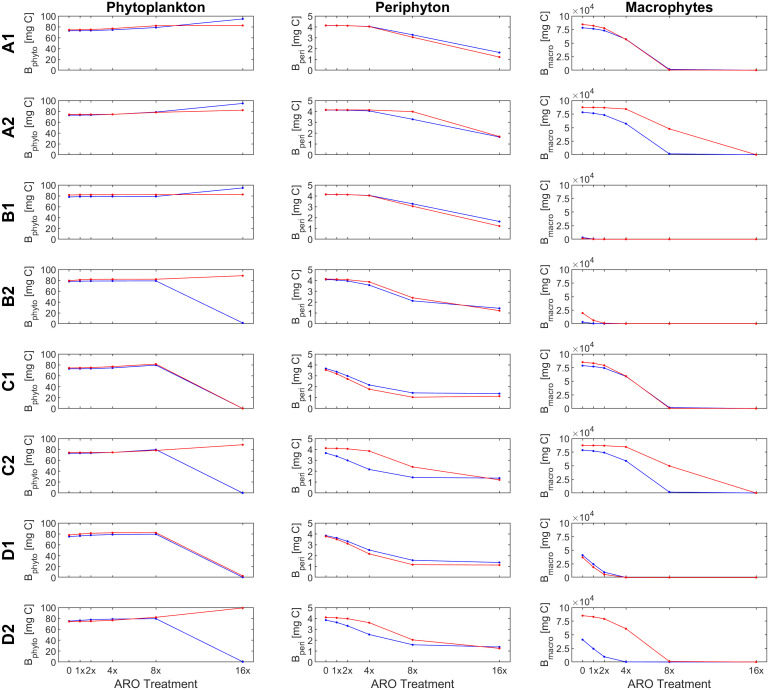
Response of primary producer groups under the (A1- D2) for the case of a single, slow-growing phytoplankton group that is highly resistant to the herbicide (simulation set 2, group *β* only) under ambient (22°C, blue) and warm (26°C, red) temperature conditions.

**Figure 6:**
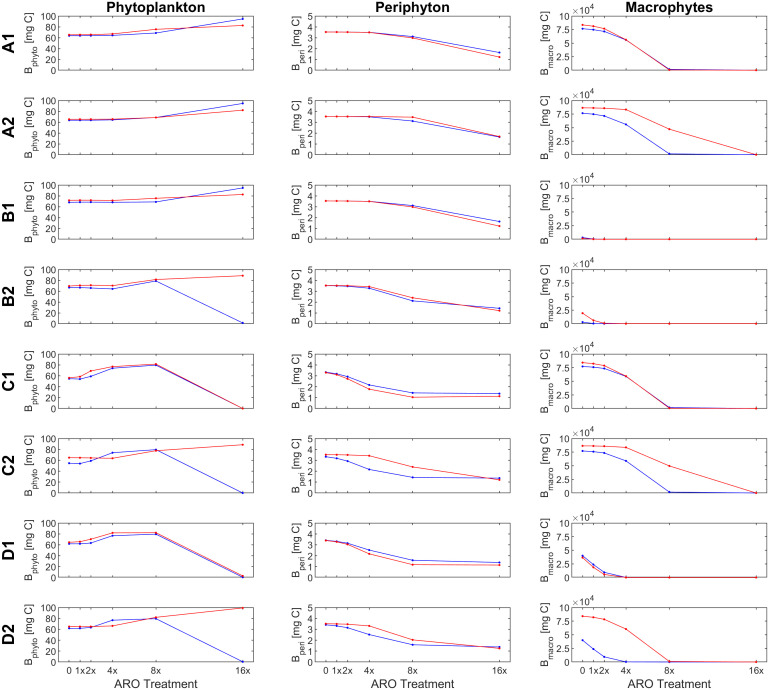
Response of primary producer groups under the eight scenarios eight scenarios (A1- D2) for the case of a mixed phytoplankton community (simulation set 3, groups *α* and *β*) under ambient (22°C, blue) and warm (26°C, red) temperature conditions.

Our custom-tailored process-based model showed that, in all simulation sets, periphyton and macrophyte carbon biomass values per unit volume (mg C m^-3^) decreased along the axis of increasing ARO concentrations at both 22°C and 26°C. Whether this decrease was more or less pronounced, and the pattern itself of the response ultimately depended on how well phytoplankton developed, with a stronger decrease in benthic primary producers when phytoplankton performed better. For simulation set 1, a community of only fast-growing phytoplankton that are highly sensitive to the herbicide (group α only, **Figure 4**), none of the eight scenarios resulted in phytoplankton response patterns similar to those observed empirically, i.e., from the microcosm experiment (**Figures 1**, **2**). Empirical and simulated phytoplankton response patterns to ARO were more comparable for simulation sets 2 (group *β* only, **Figure 5**) and 3 (mixed community of phytoplankton groups *α* and *β*, **Figure 6**), but only under warm conditions. Correlation coefficients calculated for the 3 sets also showed that the best fits across all primary producer groups were obtained for sets 2 and 3 when herbicide sensitivities were assumed to be temperature dependent and to decrease over time as a result of tolerance development, i.e., scenario D2 (**Table 5**).

**Table 5:** Correlation coefficients between simulated and observed patterns under eight scenarios and three cases of phytoplankton community composition.

Scenario	Phytoplankton		Periphyton		Macrophytes	
	Cool	Warm	Cool	Warm	Cool	Warm
Set 1: Phytoplankton group *α* only						
**A1**	*-0.514*	*-0.532*	0.582	0.876**	0.472	0.777*
**A2**	*-0.514*	*-0.524*	0.582	0.786*	0.472	0.615
**B1**	*-0.502*	*-0.552*	0.584	0.877**	0.969**	0.579
**B2**	*-0.170*	*-0.549*	0.748*	0.927**	0.969**	0.753*
**C1**	*-0.522*	*-0.381*	0.862**	0.951**	0.453	0.757*
**C2**	*-0.522*	*-0.562*	0.862**	0.926**	0.453	0.598
**D1**	0.013	*-0.416*	0.843**	0.970**	0.836**	0.849**
**D2**	0.013	*-0.547*	0.843**	0.952**	0.836**	0.765*
Set 2: Phytoplankton group *β* only						
**A1**	0.471	0.537	0.654	0.926**	0.471	0.777*
**A2**	0.471	0.506	0.654	0.798*	0.471	0.614
**B1**	0.537	0.644	0.655	0.926**	0.969**	0.579
**B2**	*-0.509*	0.660	0.805*	0.968**	0.969**	0.753*
**C1**	*-0.522*	*-0.523*	0.883**	0.869**	0.465	0.770*
**C2**	*-0.522*	0.539	0.883**	0.968**	0.465	0.606
**D1**	*-0.491*	*-0.522*	0.870**	0.915**	0.837**	0.849**
**D2**	*-0.491*	0.586	0.870**	0.979**	0.837**	0.766*
Set 3: Mixed community of phytoplankton groups *α* and *β*						
**A1**	0.477	0.518	0.590	0.878**	0.472	0.777*
**A2**	0.477	0.544	0.590	0.779*	0.472	0.615
**B1**	0.507	0.480	0.592	0.880**	0.969**	0.579
**B2**	*-0.582*	0.439	0.778*	0.951**	0.969**	0.753*
**C1**	*-0.365*	*-0.344*	0.861**	0.890**	0.459	0.767*
**C2**	*-0.365*	0.432	0.861**	0.950**	0.459	0.604
**D1**	*-0.414*	*-0.365*	0.850**	0.939**	0.837**	0.849**
**D2**	*-0.414*	0.505	0.850**	0.971**	0.837**	0.766*

* Significant at a 90% confidence level (p-value<0.1).

** Significant at a 95% confidence level (p-value<0.05).

** (in bold) Significant at a 99% confidence level (p-value<0.01).

## 4 Discussion

To better understand the complex nature of primary producer interactions and responses to multiple stressors, we combined indoor microcosm experiments with a custom-tailored process-based model. We confirmed hypotheses 1) that ARO differentially affects primary producer groups increasing the likelihood of phytoplankton dominance in shallow aquatic ecosystems and 3) that custom-tailored process-based models can support mechanistic understanding of experimental results through scenario comparison. Hypothesis 2, a facilitation of shifts to phytoplankton dominance by warming, was only partially confirmed. Here we discuss the respective implications of our findings.

### 4.1 Agricultural run-off differentially affects primary producers and their interactions

Our results demonstrate that ARO has a differential effect on the tested primary producer groups. While we did not observe a full dominance of either primary producer group at the end of the microcosm experiment, an increasing proportion of phytoplankton and decreasing proportion of macrophytes in the total microcosm biomass clearly indicates an increasing risk for the system to shift to full phytoplankton dominance. The overall non-linear increase of phytoplankton biomass with increasing ARO concentrations was most likely caused by nitrate (Scheffer et al., 1993; Olsen et al., 2015). Similar microcosm studies testing the same ARO mixture also found an increase in phytoplankton biomass with accompanied decrease in macrophyte biomass when nitrate and pesticides co-occurred (Polst et al., 2022; Vijayaraj et al., 2022). The increase in phytoplankton biomass may have contributed to the observed decline in macrophyte biomass by shading. Regime shifts with macrophyte loss due to nutrient loading are usually caused by increased shading by periphyton and phytoplankton (Phillips et al., 2016). Yet, at 22°C, a decline in macrophyte biomass was already observed at the lowest ARO concentration, which did not increase phytoplankton biomass, indicating a higher sensitivity of the tested submerged macrophyte species to ARO compared with phytoplankton. This contrasts the findings of Giddings et al. (2013) who reported a higher sensitivity of phytoplankton species compared with macrophytes for 4 out of 5 photosystem-II-inhibiting herbicides in a meta-analysis based on standardized single-species tests. In longer lasting experiments allowing for multiple generation cycles, microalgae community composition can change *via* acclimation, adaptation and selection of more tolerant species leading to a pollution-induced community tolerance (Blanck, 2002; Lorente et al., 2015; Lips et al., 2022). Such processes could explain the higher sensitivity and the lower tolerance of macrophytes to ARO in comparison to phytoplankton and periphyton in our experiment. Our results thus suggest that differential sensitivities of primary producers to pesticides can modulate their competition for light and facilitate the loss of macrophytes, eventually leading to regime shifts in shallow lentic aquatic ecosystems.

### 4.2 Warming affects the risk of an ARO-induced regime shift to phytoplankton dominance

In our experiment, ARO at both tested water temperatures clearly changed the contributions of different primary producers to the total biomass towards a higher proportion of phytoplankton indicating the risk of an ARO-induced shift to full phytoplankton dominance. However, absolute macrophyte biomass did not respond to ARO in the warming (+4°C) treatment, similar to findings by Allen et al. (2021). Possibly, higher temperatures led to an enhanced macrophyte growth partially, compensating negative pesticide effects on macrophytes. Higher growth rates of macrophytes at higher temperatures are known from other studies (e.g., Zhang et al., 2019; Zhang et al., 2022). Moreover, Vijayaraj et al. (2022) and Polst et al. (2022) described a stronger decrease in macrophyte biomass and thus lower threshold levels for ARO-induced shifts to phytoplankton dominance in +4°C treatments. These results may be caused by a lower sensitivity of phytoplankton towards herbicides at elevated temperatures (Chalifour and Juneau, 2011; Tasmin et al., 2013). Periphyton also seems less sensitive to herbicides at elevated temperatures (Larras et al., 2013), but we could not confirm this neither in our experiment nor in related experiments using the same ARO mixture (Allen et al., 2021; Polst et al., 2022). Another recent study also found no effect of combined warming, nutrients and pesticides on periphyton and suggested that treatment effects compensated each other (Zhang et al., 2022). We conclude that warming facilitates macrophyte and phytoplankton growth, which can modulate macrophyte response thresholds to ARO. However, the increasing proportion of phytoplankton in the primary producer community under combined warming and ARO exposure indicates an overall increased risk of macrophyte loss, also supported by findings in Vijayaraj et al. (2022) and Polst et al. (2022).

### 4.3 Process-based models support mechanistic understanding of experimental results

Custom-tailored models of appropriate complexity, i.e., including only the processes that are strictly necessary to reproduce empirical observations, are helpful to shed light on the critical mechanisms behind ecosystem-wide responses (Vasconcelos et al., 2016; López Moreira et al., 2021). This is particularly true when addressing the effects of multiple stressors, for which the knowledge base is currently just starting to develop. Moreover, the nearly infinite amount of combinations of stressor levels limits the potential contribution of purely laboratory-based studies, e.g., microcosm experiments, where decisions are made based on several factors including space, materials and budget available, viability and phenology of model organisms, time constraints and the human effort that is needed. Considering that readily available models would need substantial modifications, recalibration and revalidation before use and may still remain unnecessarily complex for exploratory research, we suggest to calibrate and validate newly developed, custom-made simple models with experimental data and to test scenarios which allow subsequent model refinements and mechanistic interpretation of model results.

Our pattern-oriented modeling effort allowed for the reconstruction of primary producer temporal dynamics at different levels of approximation to the empirical observations. It also made it possible to assess the trajectories of all state variables (exemplified in **Figures S3**, **S4**) and the most likely limiting conditions to primary producer growth and their change over time (exemplified for epiphyton in **Figure S5**). Because the empirical data was mostly limited to end-point values, however, model calibration and scenario selection was limited to final biomass results. Correlation coefficients allowed for the quantitative identification and selection of the most plausible scenario that best fitted empirical observations.

#### 4.3.1 Phytoplankton biomass

Assuming a community of only fast-growing phytoplankton that are highly sensitive to the herbicide (set 1: group *α* only), correlation coefficients for phytoplankton biomass where mostly negative (**Table 5**), indicating that the model was clearly not able to reproduce the pattern observed in the experiment, where final biomass values were generally higher for the highest ARO concentrations (**Figures 2**, **4**). This complex phytoplankton response was better captured under the assumption of a single, slow-growing but relatively resistant phytoplankton group (set 2: group *β* only) (**Figure 5**) and under the assumption of a mixed phytoplankton community (set 3: both groups) (**Figure 6**), for which we obtained several positive albeit not statistically significant correlation coefficients (**Table 5**). However, this was generally true only under warm conditions (26°C). The exceptions to this were scenarios C1 and D1, for which correlation coefficients for phytoplankton were negative under both temperature treatments (both sets, although marginally less negative for set 3, mixed phytoplankton community).

#### 4.3.2 Periphyton and macrophyte biomass

For periphyton, statistically significant positive correlations were obtained for all three sets under all eight scenarios at the higher temperature of 26°C (**Table 4**). For the cool temperature treatment (22°C), these positive correlations were statistically significant only under scenarios B2, C1-2 and D1-2. Macrophyte responses to ARO were also generally positively correlated across all scenarios, sets and temperature treatments, but statistically significant only under scenarios B1-2 and D1-2 at 22°C, and under scenarios B2, C1 and D1-2 at 26°C (**Table 4**). However, the only scenario where the simulated macrophyte response pattern qualitatively resembled the observed one was scenario D2 (**Figure 6**).

#### 4.3.3 Most plausible scenario

To identify the most plausible scenario, we first discarded all scenarios where final macrophyte biomass patterns were not significantly correlated under both temperature treatments. Scenarios A1-2, B1, and C1-2 were thus removed from further consideration. Subsequently, scenario D1 was also discarded, as it failed to produce positive correlations for phytoplankton biomass. Finally, considering that, between the two remaining scenarios, i.e., scenarios B2 and D2: a) phytoplankton responses were both qualitatively and quantitatively fundamentally the same under scenarios B2 and D2; b) positive correlations for periphyton were higher and more statistically significant under scenario D2; and that, c) qualitatively, macrophytes were best simulated under scenario D2, quantitatively supported by statistically significant and highly positive correlation coefficients; we identified and selected scenario D2 as the most plausible scenario.

#### 4.3.4 Comparisons among the three simulation sets

Because the model failed to produce good fits for the final phytoplankton biomass response pattern for set 1 (group *α* only), with all correlation coefficients being negative at 26°C and only marginally positive for scenarios D1-2 at 22°C (**Table 5**), this simulation set was removed from any further consideration.

Under scenario D2, selected as the most plausible one, for simulation sets 2 (group *β* only) and 3 (mixed community of groups *α* and *β*), simulated and observed responses were positively correlated for both periphyton (lowest correlation coefficients were 0.870 and 0.850 for sets 2 and 3 at 22°C, respectively) and macrophytes (lowest correlation coefficients were 0.766 for both sets 2 and 3 at 26°C). All these correlations were statistically significant at a confidence level of either 90%, 95% or even 99% (**Table 5**). Phytoplankton responses, however, were only weakly positively correlated in the warming treatment (0.586 and 0.505 for sets 2 and 3 at 26°C, respectively) and even negatively correlated under ambient temperature (22°C). However, none of these correlations for phytoplankton were statistically significant.

In scenario D2, sensitivities to the herbicide of all primary producer groups were dependent on temperature (organisms were less sensitive at the higher temperature). Additionally, herbicide sensitivities of all microscopic primary producers also decreased over time as they became more tolerant to the herbicide. This resulted in a simulated pattern under scenario D2 that was positively correlated with the observed phytoplankton response for the warm temperature treatment. Under scenario D1, where sensitivities were not affected by temperature, a positive correlation could not be achieved for any of the sets at neither of the two temperature treatments.

In our study, the most plausible scenario was the one involving stress-induced phytoplankton tolerance development, including temperature-dependent organism acclimation and adaptation to ARO (scenario D2) as well as stress-induced succession in communities (simulation set 3, mixed phytoplankton community). The relevance of these processes has been shown under the concept of pollution-induced community tolerance (Blanck, 2002; Schmitt-Jansen et al., 2016; Lips et al., 2022). Another recent study reported observed changes in phytoplankton communities of small lentic waters following exposure to agricultural run-off (Wijewardene et al., 2021) but did not test for changes in tolerance to the pesticides. While designed for a specific experimental set-up, our model can be used for future hypothesis testing in experimental studies with a similar complexity (focusing on primary producer interactions), but can also be extended to include higher trophic levels.

### 4.4 Final remarks

Our microcosm experiment revealed a differential response of aquatic primary producers to the combined effects of warming and ARO, potentially leading to phytoplankton dominance, a less desirable ecosystem state. At both tested temperatures, phytoplankton was favored and increased its biomass and proportion in the primary producer community with increasing ARO concentrations due to the higher availability of nitrate and ability to adapt to pesticides. Macrophytes became less sensitive to ARO with warming, but may still be weakened due to the increasing proportion of phytoplankton eventually shading them out. The development of a simple, process-based model allowed understanding the role of temperature dependence, tolerance development and community adaptation to combined ARO and warming, because a scenario including these processes ultimately led to the best fit between predicted and observed responses. Our results highlight the importance of considering stress-induced tolerance development, adaptation and succession when predicting mid- and long-term effects of toxicants on complex primary producer communities that include multiple species. Trait diversity may compensate for the direct negative effects of pesticides on individual species to keep ecosystems productive at a macroscopic scale, especially at higher temperatures and under nutrient-enriched conditions, where ARO may unbalance the system through indirect effects on vulnerable communities like macrophytes.

## Data availability statement

The raw data supporting the conclusions of this article will be made available by the authors, without undue reservation.

## Author contributions

The experimental part of the study was mostly done by BP. SH and other colleagues at the IGB Berlin offered help during the final sampling activities of the experiment. The process-based model was developed and implemented by GLMM. SH, FH, MS-J, and BP all provided guidance for the development of the model. GLMM and BP contributed equally to the final manuscript. EG helped setting up the experiment, provided guidance and contributed to the discussion and editing of the manuscript as Principal Investigator of the CLIMSHIFT project, which funded this work. All authors contributed to the article and approved the submitted version.
