# Supplementary material for: Microcosm experiment combined with process-based modeling reveals differential response and adaptation of aquatic primary producers to warming and agricultural run-off
Source: Front Plant Sci. 2023 Jun 19;14:1120441. doi: 10.3389/fpls.2023.1120441 (PMC10316517; doi:10.3389/fpls.2023.1120441)
Supplement: Supplementary file 1 [file DataSheet_1.docx]

**SUPPLEMENTARY INFORMATION**

**Microcosm experiment combined with process-based modeling reveals differential response and adaptation of aquatic primary producers to warming and agricultural run-off**

Gregorio A. López Moreira Mazacotte, Bastian H. Polst, Elisabeth M. Gross, Mechthild Schmitt-Jansen, Franz Hölker and Sabine Hilt

**Microcosm geometry**

For modeling purposes, we modeled the microcosms as right cylindrical vases of diameter $\varphi_{V}= 2.500\times{10}^{-1}$ m and height $H_{V}= 4.000\times{10}^{-1}$ m. The resulting base area $A_{base}= 4.910\times{10}^{-2}$ m^2^, lateral area is $A_{lat}= 3.142\times{10}^{-1}$ m^2^ and volume is of $V= 1.96\times{10}^{-2}$ m^3^. Wall periphyton was assumed to grow on the vertical inner glass surface of the vases only, so that $A_{peri}=A_{lat}$.

**Temporal settings**

All simulations were run with an hourly time step for a total of 19 days, coinciding with the duration of the experiment, ending at the start of macrophyte sampling, when the lights were turned off.

**Concentration of toxicants and nutrients in the ARO mix**

The following concentrations of toxicants were adopted for the standard the ARO mix (1x):

- Terbuthylazine (herbicide): 0.75 µg L^-1^
- Pirimicarb (insecticide): 3.75 µg L^-1^
- Tebuconazole (fungicide): 22.5 µg L^-1^
- Copper (as copper(II) sulfate): 10.5 µg L^-1^
- Nitrate (as potassium nitrate): 2250 µg L^-1^

Multiples of the standard ARO mix (1x) were applied following a geometric sequence with enrichment factor of two (2), i.e., 2x, 4x, 8x and 16x, keeping proportions among all components constant. Since the experiment focused on primary producers, only the herbicide terbuthylazine (TBA) was included as toxicant in the simulations.

**Boundary conditions**

*Incident Photosynthetically Active Radiation (PAR)*

A 16h:8h light:dark daily cycle was applied to the microcosms, with a constant incident PAR of ~16.8296 W m^-2^ for the lit period, estimated to be equivalent to the 77.2 µmol m^-2^ s^-1^ (mean value) measured at the water surface during the experiment. For this, the following conversion factor was used: 1 µmol m^-2^ s^-1^ ≈ 0.218 W m^-2^ for Photosynthetically Active Radiation (PAR, 400-700 nm) originating from a cool white fluorescent light source (Sager and McFarlane, 1997).

*Water temperature*

Microcosms were assumed to maintain constant temperatures of $T_{w,cool}=22$°C and $T_{w,warm}=26$°C for the cool and warm treatments, respectively, as in the experiment.

*Live and dead phytoplankton biomass, bound organic phosphorus and dissolved inorganic phosphorus concentrations*

The model was set so that no living or dead phytoplankton cells entered the microcosms from outside the system. Leaving the water column was allowed only through the bottom, resulting from phytoplankton sinking and eventually settling into the sediments, carrying within them the amounts of organic phosphorus given by their fixed phosphorus-to-carbon cellular quota. Phytoplankton was considered to instantly die when reaching the sediment layer. No direct inflows or outflows of dissolved inorganic phosphorus from external sources were permitted aside from the regular nutrient additions, as described next.

*Nutrient additions*

To reflect experimental conditions (a mixture of KNO_3_ and KH_2_PO_4_ was added to the microcosms twice a week), four scheduled nutrient additions were programmed to happen during the simulation runs on days 4, 8, 12 and 16. The concentrations of phosphorus and nitrogen in these periodic additions followed the Redfield ratio (Redfield, 1958), and were: 30.974 µg L^-1^ and 224.11 µg L^-1^., respectively, equivalent to 1 µmol L^-1^ and 16 µmol L^-1^, respectively.

**Auxiliary equations**

In this section, we present the formulation of all auxiliary variables appearing in the governing equations of the model, hereafter referred to as primary auxiliary variables, as well as the formulation of secondary auxiliary variables, i.e., those appearing in the equations of primary auxiliary variables. All parameters appearing in these equations are described in Tables S1-S6, where we also present the chosen values and list relevant references to justify our choices.

*Light extinction*

The extinction of shortwave radiation energy absorbed into the system through the water surface is modeled following the Beer-Lambert law. At any given depth $z$, we have

|  | $\frac{dI_{sw}}{dz}=-k_{tot}I_{sw},$ | $\left( S1 \right)$ |
| --- | --- | --- |

where $k_{tot}$ is the total light extinction coefficient resulting from adding the individual contributions of live and dead carbon biomass of both phytoplankton groups, $k_{live,phyto}$ and $k_{dead,phyto}$, respectively, to that which is due to water and solutes therein, $k_{bg}=0.3 m^{-1}$ (assumed value in the range of those used in similar studies, e.g., Jäger et al., 2010). The total light extinction coefficient is therefore given by

|  | $k_{tot}=k_{live,phyto}+k_{dead,phyto}+k_{bg}$, | $\left( S2 \right)$ |
| --- | --- | --- |

where

|  | $k_{live,phyto}=\sum_{g=A,B} k_{live,spec,{phyto}_{g}}C_{live,{phyto}_{g}}$ | $\left( S3 \right)$ |
| --- | --- | --- |

and

|  | $k_{dead,phyto}=\sum_{g=A,B} k_{dead,spec,{phyto}_{g}}C_{dead,{phyto}_{g}}$ | $\left( S4 \right)$ |
| --- | --- | --- |

$k_{live,spec,{phyto}_{g}}$ and $k_{dead,spec,{phyto}_{g}}$ being, respectively, the specific, i.e., per unit concentration, light extinction coefficients of live and dead carbon biomass of phytoplankton group $g=A,B$.

From eq. S1, the Beer-Lambert law predicts that, at any given depth $z$,

|  | $I_{sw}\left( z \right)=I_{sw,surf}e^{-k_{tot}z}$, | $\left( S5 \right)$ |
| --- | --- | --- |

where $I_{sw,surf}$ is the shortwave irradiance at the water surface.

*Light availability per primary producer group*

1. Phytoplankton and periphyton

Because the experiment started with short macrophyte shoots and ran for a relatively short period, we assumed no influence of macrophytes on the light that was available to phytoplankton or periphyton growing on the glass surfaces of the microcosms. The one-box model set-up required us to determine a mean value of the PAR function produced by the Beer-Lambert law. $I_{PAR,mean,phyto}$ and $I_{PAR,mean,peri}$, the PAR that was available to phytoplankton (both groups) and periphyton were therefore calculated as

|  | $I_{PAR,mean,phyto}=\frac{I_{PAR,surf}}{k_{tot}H_{V}}\left( 1-e^{-k_{tot}H_{V}} \right)$, | $\left( S6a \right)$ |
| --- | --- | --- |

and

|  | $I_{PAR,mean,peri}=\frac{I_{PAR,surf}}{k_{tot}H_{V}}\left( 1-e^{-k_{tot}H_{V}} \right)$, | $\left( S6b \right)$ |
| --- | --- | --- |

where $I_{PAR,surf}$, is the fraction of the incident PAR, $I_{PAR,in}=16.8296 W m^{-2}$, that remains at the water surface after subtracting that which is reflected back out. This remaining fraction is calculated as

|  | $I_{PAR,surf}=\left( 1-r_{S} \right)I_{sw,in}$, | $\left( S7 \right)$ |
| --- | --- | --- |

where $r_{S}$ is the albedo of the microcosm water. We assumed this albedo to be $r_{S}=0.04$, so that, as per eq. S7, $I_{sw,surf}=\left( 1-0.04 \right)\times16.8296 W m^{-2}=16.1564 W m^{-2}$ .

1. Macrophytes and epiphyton

Macrophyte biomass also contributes to the extinction of light along the water column. We assumed this contribution to light extinction to be dependent on the dynamic macrophyte height. This dynamic height is computed, for every time step, from instant macrophyte carbon biomass based on an assumed constant height-to-carbon biomass ratio, ${HC}_{ratio,macro}$, as follows:

|  | $H_{macro}=C_{live,macro}{HC}_{ratio,macro}$. | $\left( S8 \right)$ |
| --- | --- | --- |

Light is extinguished as it penetrates the water column so that, following eq. S1b, PAR reaching the top of the macrophyte layer can be calculated as

|  | $I_{PAR,macro,top}=I_{PAR,surf}e^{-k_{tot}\left( H_{V}-H_{macro} \right)}$. | $\left( S9 \right)$ |
| --- | --- | --- |

Nevertheless, epiphyton ‘intercepts’ and absorbs a fraction of the light that would otherwise be fully available to macrophytes. Köhler et al. (2010), found that total epiphyton reduced incoming shortwave radiation available to macrophytes by as much as 61% under sunny conditions and by as much as 71% under shaded conditions, and proposed the following empirical relationship between $a_{epi}$, the absorbed fraction, and ${DW}_{epi}$, the dry weight of epiphyton per unit area:

|  | $a_{epi}=1-e^{-0.083{DW}_{epi}}$. | $\left( S10a \right)$ |
| --- | --- | --- |

To formulate an equivalent relationship as a function of carbon biomass, we first converted ${DW}_{epi}$ to ${WW}_{epi}$, the wet weight of epiphyton biomass, based on the results by Sládeček & Sládečková (1964), which reported a mean dry-to-wet weight ratio of ~0.0807, so that eq. S10a becomes:

|  | $a_{epi}=1-e^{-0.083\times0.0807{WW}_{epi}}$. | $\left( S10b \right)$ |
| --- | --- | --- |

We then converted ${WW}_{epi}$ to $C_{epi}$, the total carbon biomass of epiphyton, based on the rough assumption that at least 99% of the weight of algal cells is water (Wiley, et al., 2011) and that the remaining 1% follows the C:N:P = 1 273.124 : 224.110 : 30.974 mass-based Redfield ratios that correspond to the usual C:N:P = 106:16:1 atom-based ratios. For this, we neglected the contribution of all other elements to the total epiphyton biomass, i.e., we assumed that all non-water biomass was made up of carbon, nitrogen and phosphorus only. Under this assumption, 1 273.1236 units of carbon biomass correspond to 224.1097 units of nitrogen biomass and 30.97376 units of phosphorus biomass, adding up to 1 528.20704 units of non-water biomass (1% of ${WW}_{epi}$), so that total wet weight would be 152 820.704 units. Thereby, the following relationship between wet weight and carbon biomass can be established:

|  | ${WW}_{epi}=\frac{152 820.704}{1 273.1236}C_{epi}=120.036031C_{epi}$, | $\left( S11 \right)$ |
| --- | --- | --- |

so that

| $a_{epi}=1-e^{-0.083\times0.0807\times120.036031C_{epi}}=1-e^{-0.083\times9.687C_{epi}}$. | $\left( S10c \right)$ |
| --- | --- |

Finally, by making $C_{epi}=C_{live,epi}+C_{dead,epi}$, we obtained the following relationship:

|  | $\alpha_{epi}=1-e^{-{shad}_{epi}\times9.687\left( C_{live,epi}+C_{dead,epi} \right)}$. | $\left( S10d \right)$ |
| --- | --- | --- |

where ${shad}_{epi}=0.083$ as in Köhler et al. (2010).

Below the top of the macrophyte layer, self-shading by macrophytes leads to a further attenuation of light that is available to them. Similarly, to what we did for phytoplankton and periphyton, we calculated the mean value of light that is available to macrophytes as

|  | $I_{PAR,mean,macro}=\left( 1-\alpha_{epi} \right)\frac{I_{PAR,macro,top}}{k_{tot,macro}H_{macro}}\left( 1-e^{-k_{tot,macro}H_{macro}} \right)$, | $\left( S12 \right)$ |
| --- | --- | --- |

where $k_{tot,macro}$ is the increased light extinction coefficient for the dynamic macrophyte layer, calculated as

|  | $k_{tot,macro}=k_{tot}+k_{live,macro}C_{live,macro}+k_{dead,macro}C_{dead,macro}$, | $\left( S13 \right)$ |
| --- | --- | --- |

$k_{live,macro}$ and $k_{dead,macro}$ being the specific light extinction coefficients of live and dead macrophyte carbon biomass, respectively. Finally, because epiphyton growth is limited to the extent of the macrophyte layer, we have that the mean value of PAR that epiphyton receives can be calculated as

|  | $I_{PAR,mean,epi}=\frac{I_{PAR,macro,top}}{k_{tot,macro}H_{macro}}\left( 1-e^{-k_{tot,macro}H_{macro}} \right)$. | $\left( S14 \right)$ |
| --- | --- | --- |

Fractional growth rates

1. Phytoplankton

To account for the dependence of phytoplankton biomass growth on water temperature, PAR availability and phosphorus concentration (as the limiting nutrient), the fractional growth rate of phytoplankton carbon biomass was modeled as

|  | $p_{{phyto}_{g}}=\mu_{ref,{phyto}_{g}}{TMF}_{{phyto}_{g}}\min\left( {LLF}_{{phyto}_{g}},{PLF}_{{phyto}_{g}} \right)$, | $\left( S15 \right)$ |
| --- | --- | --- |

where $\mu_{ref,{phyto}_{g}}$ is a reference fractional growth rate of carbon biomass of phytoplankton; ${TMF}_{{phyto}_{g}}$ is an Arrhenius-type temperature-mediation factor; ${LLF}_{{phyto}_{g}}$ is a light limitation factor accounting for photoinhibition of phytoplankton growth (Steele, 1962); and ${PLF}_{{phyto}_{g}}$ is a Monod-type phosphorus limitation factor, all potentially specific to each phytoplankton group $g=\alpha,\beta$. These are:

|  | ${TMF}_{{phyto}_{g}}={\theta_{p_{{phyto}_{g}}}}^{\left( T_{w,tr}-T_{w,ref,bio} \right)}$, | $\left( S16 \right)$ |
| --- | --- | --- |

where $\theta_{p_{{phyto}_{g}}}$ is the corresponding pre-exponential factor, also known as the temperature constant, $T_{w,tr}$ is the water temperature for either the cool or warm treatment, and $T_{w,ref,bio}$ the reference water temperature for biological processes;

|  | ${LLF}_{{phyto}_{g}}=\frac{I_{sw}}{I_{sw,opt,{phyto}_{g}}}e^{\left( 1 - \frac{I_{sw}}{I_{sw,opt,{phyto}_{g}}} \right)}$, | $\left( S17 \right)$ |
| --- | --- | --- |

where $I_{sw,opt,{phyto}_{g}}$ is the optimal PAR value for the growth of phytoplankton group $g=\alpha,\beta$; and

|  | ${PLF}_{{phyto}_{g}}=\frac{P_{d}}{m_{P,{phyto}_{g}}+P_{d}}$, | $\left( S18 \right)$ |
| --- | --- | --- |

where $m_{P,{phyto}_{g}}$ is the half-velocity constant of the Monod-type phosphorus limitation factor for phytoplankton group $g=\alpha,\beta$. Following Liebig’s law of the minimum, i.e., that growth is limited by the scarcest resource, we take the growth of each phytoplankton group to be limited by the minimum value between ${LLF}_{{phyto}_{g}}$ and ${PLF}_{{phyto}_{g}}$.

1. Periphyton and epiphyton

As for the case of phytoplankton, periphyton and epiphyton growth depends on water temperature, PAR availability and phosphorus concentration. However, because these primary producers need a surface to grow on, which is not infinite and becomes limiting as biomass densities approach a maximum value after which they naturally detach, we introduced an additional limiting factor that applies to both periphyton and epiphyton growth: a space limitation factor, ${SLF}_{i=peri,epi}$, so that

|  | $p_{peri}=\mu_{ref,peri}{TMF}_{peri}\min\left( {LLF}_{peri}{,PLF}_{peri},{SLF}_{peri} \right)$ | $\left( S19 \right)$ |
| --- | --- | --- |

and

|  | $p_{epi}=\mu_{ref,epi}{TMF}_{epi}\min\left( {LLF}_{epi},{PLF}_{epi},{SLF}_{epi} \right)$, | $\left( S20 \right)$ |
| --- | --- | --- |

where the $TMF$,$LLF$ and$PLF$ factors modifying the reference growth rates are analogous to those for phytoplankton (eqs. S15-S17).

The space limitation factors are Monod-type factors formulated as

|  | ${SLF}_{peri}=\frac{A_{peri}}{m_{Sp,peri}+A_{peri}}$ | $\left( S21 \right)$ |
| --- | --- | --- |

and

|  | ${SLF}_{peri}=\frac{A_{epi}}{m_{Sp,epi}+A_{epi}}$, | $\left( S22 \right)$ |
| --- | --- | --- |

where, $m_{Sp,peri}$ and $m_{Sp,epi}$ are the half-velocity constants, $A_{peri}$ is the constant area on which periphyton can grow, which is equal to the lateral area of the vase, i.e.,

|  | $A_{peri}=\pi\varphi_{V}H_{V}$, | $\left( S23 \right)$ |
| --- | --- | --- |

and $A_{epi}$ is the variable area on which epiphyton can grow, i.e., the dynamic area of living and dead macrophyte surfaces, calculated as

|  | $A_{epi}={AC}_{ratio,macro}\left( C_{live,macro}+C_{dead,macro} \right)$, | $\left( S24 \right)$ |
| --- | --- | --- |

where ${AC}_{ratio,macro}$ is an assumed area-to-carbon biomass rate for macrophytes.

1. *Macrophytes*

Unlike phytoplankton, periphyton and epiphyton, we assumed the growth of macrophytes not to be limited by phosphorus, as the initial biomass in each pot was quite small and the sub-experiment ran only for a short time. The fractional macrophyte growth rate is therefore only influenced by water temperature and available PAR, so that

|  | $p_{macro}=\mu_{ref,macro}{TMF}_{macro}{LLF}_{macro},$ | $\left( S25 \right)$ |
| --- | --- | --- |

where $\mu_{ref,macro}$ is a reference fractional growth rate of macrophyte biomass; and ${TMF}_{macro}$ and ${LLF}_{macro}$ are the temperature mediation and light limitation factors for macrophytes, respectively, both of which were calculated differently for macrophytes than for the other three groups of primary producers.

On the one hand, the Arrhenius-type temperature mediation factor was replaced by a temperature optimum curve (as in Hilt, et al., 2018). This is:

|  | ${TMF}_{macro}=e^{-\frac{0.5}{{\sigma_{T,macro}}^{2}}\left[ \left( T_{w,tr}-T_{w,opt,macro} \right)^{2}-\left( T_{w,ref,bio}-T_{w,opt,macro} \right) \right]}$, | $\left( S26 \right)$ |
| --- | --- | --- |

$\sigma_{T,macro}$ being a temperature constant based on a Gaussian curve and $T_{w,opt,macro}$ the optimal water temperature for macrophyte growth. On the other hand, given that macrophyte light requirements are generally much higher for macrophytes (Sand-Jensen & Borum, 1991), we considered photoinhibition not to be a potential issue and therefore used a Monod-type formulation of light limitation for *P. pectinatus* as implemented in the SAGA model (Hootsmans, 1994). This is:

|  | ${LLF}_{macro}=\frac{I_{PAR,mean,macro}}{h_{macro}+I_{PAR,mean,macro}},$ | $\left( S27 \right)$ |
| --- | --- | --- |

where $h_{macro}$ is the half-saturation constant.

Fractional loss rates (all groups)

Because primary consumers were absent in the experiment, no grazing was implemented. Therefore, primary producers lose carbon biomass mainly due to cellular death and other intrinsic processes such as exudation and respiration (which we here neglected for the sake of simplicity). To account for all these, we modeled the fractional loss rate of primary producer biomass as

|  | $l_{i}=l_{d,i}+l_{exu,i}$, | $\left( S28 \right)$ |
| --- | --- | --- |

where $l_{d,i}$ and $l_{exu,i}$ are the effective death and exudation loss rates of primary producer group $i={phyto}_{g=\alpha,\beta}, peri, epi, macro$, each one formulated as a function of temperature:

|  | $l_{d,i}=l_{d,ref,i}{\theta_{l_{d,i}}}^{\left( T_{w,tr}-T_{w,ref,bio} \right)}$, | $\left( S29 \right)$ |
| --- | --- | --- |

and

|  | $l_{exu,i}=l_{exu,ref,i}{\theta_{l_{exu,i}}}^{\left( T_{w,tr}-T_{w,ref,bio} \right)}$. | $\left( S30 \right)$ |
| --- | --- | --- |

In eq. S29, $l_{d,ref,i}$ is the reference death rate, i.e., the death rate at $T_{w,ref,bio}$ (reference temperature for biological processes, 20°C). This death rate incorporates the effect of terbuthylazine, if present, and is given by a log-logistic dose-response curve with formula:

|  | $l_{d,ref,i}\left( t \right)=l_{d,min,i}+\left( l_{d,max,i}-l_{d,min,i} \right)\left[ \frac{1}{1+e^{-k_{TBA,i}\left( {TBA}_{d}\left( t \right)-{EC50}_{TBA,i} \right)}} \right]^{a_{TBA,i}}$, | $\left( S31 \right)$ |
| --- | --- | --- |

where $a_{TBA,i}>0$ and $k_{TBA,i}>0$ are positive parameters that control the shape of the curve, ${EC50}_{TBA,i}$ is the half maximal effective concentration of terbuthylazine in the dose-response relationship of group $i$, and ${TBA}_{d}$ is the dynamic volume concentration of terbuthylazine dissolved in microcosm water. The value of $l_{d,ref,i}$ tends to $l_{d,min,i}$ (minimum value) as ${TBA}_{d}$ tends to zero, and tends to $l_{d,max,i}$ (maximum value) as ${TBA}_{d}$ tends to infinity. For simplicity, we have chosen $l_{d,max,i}$ to be equal to 1.

In this study, we chose $a_{TBA,i}=1.01 \forall i$ and $k_{TBA,i}=1.10 \forall i$ as shape parameters of all dose-response curves. In scenarios D1 and D2, however, to account for the development of tolerance to the toxicant over time as a result of adaptation and or acclimation, we formulated each ${EC50}_{TBA,i}$ as a group- and scenario-specific dynamic parameter that increases over time until it reaches a maximum value, ${EC50}_{TBA,max,i}$, so that it is a multiple of the initial value ${EC50}_{TBA,ini,i}$, resulting from its multiplication by a chosen amplification factor, $f_{TBA,i}$, so that

|  | ${EC50}_{TBA,i,max}=f_{TBA,i}{EC50}_{TBA,i,ini}$. | $\left( S32 \right)$ |
| --- | --- | --- |

We present all ${EC50}_{TBA,ini,i}$ and $f_{TBA,i}$ values for all eight scenarios in Table S6.

The dynamic ${EC50}_{TBA,i}$ value is formulated as

$${EC50}_{TBA,i}\left( t \right)={EC50}_{TBA,max,i}-\left( {EC50}_{TBA,max,i}-{EC50}_{TBA,ini,i} \right)e^{-\left( 1-r_{TBA,i} \right)t}$$

|  |  | $\left( S33 \right)$ |
| --- | --- | --- |

where $r_{TBA,i}$ is a group-specific adaptation rate. This formulation leads to the desired behavior that ${EC50}_{TBA,i}\left( 0 \right)={EC50}_{TBA,ini,i}$ and $\lim_{t\to\infty} {EC50}_{TBA,i}\left( t \right)={EC50}_{TBA,max,i}$. Note that for $r_{TBA,i}=1$, ${EC50}_{TBA,i}\left( t \right)={EC50}_{TBA,ini,i}$, i.e., there is no adaptation over time. This was the case for all microscopic primary producer groups under scenarios A1-2, B1-2 and C1-2, as well as for macrophytes in all eight scenarios. In scenarios D1 and D2, however, we made $r_{TBA,i}=0.99$ for all microscopic primary producers.

Fractional biodegradation rates of dead biomass and cellular exudates

In all ecosystems, all organic substances, dead biomass and cellular exudates included, are ultimately biodegraded/re-mineralized by the decomposers (e.g., bacteria and fungi). Several factors affect the rate at which biodegradation occurs, among which we can mention temperature, light, water and oxygen. In this model, since we are modeling the dead biomass and exudates of all primary producer groups, we have included this process by means of fractional biodegradation rates, $b_{dead,i}$ and $b_{exu,i}$ ($i=phyto,g, peri, epi, macro;g=\alpha,\beta$).

For the sake of simplicity, and because this study focuses on the effects of temperature, we have made these rates depend only on water temperature. This dependence takes the form of an Arrhenius-type function, which is:

|  | $b_{dead,i}=b_{dead,ref,i}{\theta_{b_{dead,i}}}^{\left( T_{w,tr}-T_{w,ref,bio} \right)}$ | $\left( S34 \right)$ |
| --- | --- | --- |

and

|  | $b_{exu,i}=b_{exu,ref,i}{\theta_{b_{exu,i}}}^{\left( T_{w,tr}-T_{w,ref,bio} \right)}$ | $\left( S35 \right)$ |
| --- | --- | --- |

where the $b_{dead,ref,i}$ and $b_{exu,ref,i}$ are reference fractional biodegradation rates that can be made specific to each group $i$; $\theta_{b_{dead,i}}$ and $\theta_{b_{exu,i}}$ are pre-exponential factors for biodegradation of dead biomass and cellular exudates, respectively, that can also be specific to each group; and $T_{w,ref,bio}$, a reference water temperature for biological processes, usually set to be 20°C (e.g., Sand-Jensen & Borum, 1991).

Source/sink of phosphorus terms in the phosphorus concentration differential equation

The terms appearing in the governing equation for dissolved phosphorus concentration are given by

|  | $S_{{phyto}_{g}}=b_{dead,{phyto}_{g}}q_{P,{phyto}_{g}}C_{dead,{phyto}_{g}}+b_{dead,{phyto}_{g}}q_{P,{phyto}_{g}}\frac{C_{dead,sed,{phyto}_{g}}}{H_{V}}+b_{exu,{phyto}_{g}}q_{P,{phyto}_{g}}C_{exu,{phyto}_{g}}-p_{{phyto}_{g}}q_{P,{phyto}_{g}}C_{live,{phyto}_{g}};g=\alpha,\beta$ | $\left( S36 \right)$ |
| --- | --- | --- |
|  | $S_{peri}=b_{dead,peri}q_{P,peri}C_{dead,peri}\frac{A_{peri}}{V}+b_{exu,peri}q_{P,peri}C_{exu,peri}-p_{peri}q_{P,peri}C_{live,peri}\frac{A_{peri}}{V}$ | $\left( S37 \right)$ |
|  | $S_{epi}=b_{dead,epi}q_{P,epi}C_{dead,epi}\frac{A_{epi}}{V}+b_{exu,epi}q_{P,epi}C_{exu,epi}-p_{epi}q_{P,epi}C_{live,epi}\frac{A_{epi}}{V}$ | $\left( S38 \right)$ |
|  | $S_{macro}=b_{dead,macro}q_{P,macro}\frac{C_{dead,macro}}{V}+b_{exu,macro}q_{P,macro}C_{exu,macro}$ | $\left( S39 \right)$ |

Note that, in the equation for $S_{macro}$, the phosphorus source/sink that is attributable to macrophytes (eq. S39), there is no sink term on the right-hand side because we considered macrophytes to extract phosphorus exclusively from the sediments, the phosphorus content of which we assumed to be unlimited because the experiment only ran for a short period.

*Decay of terbuthylazine*

For the sake of simplicity, we assumed a linear decay of ${TBA}_{d}$, the volume concentration of dissolved terbuthylazine, based on experimental data (Table 4). We formulated this variable as

|  | ${TBA}_{d}\left( t \right)=\left( 1-{TBA}_{decay}t \right){TBA}_{d,ini}$, | $\left( S40 \right)$ |
| --- | --- | --- |

where $t$ is the time coordinate, ${TBA}_{d,ini}$ is the initial concentration of terbuthylazine in the microcosm water and ${TBA}_{decay}$ its fractional rate of decay.

**Initial conditions**

For all scenarios, the model was initialized with the initial biomass values shown in Table S7, as well as nutrient and toxicant concentrations in the microcosm water based on data from the experiment (Table 4).

**Mass balance**

Dynamic mass balance checks have been implemented for both carbon and phosphorus, accounting for all gain and loss processes. This allowed us to make sure that carbon and phosphorus mass is conserved throughout the simulations, as shown in Figure S6.

**Figure S1.** Effect of terbuthylazine for two species of phytoplankton, other microscopic primary producers (wall periphyton and epiphyton) and macrophytes at the beginning (top row) and by the end (bottom row) of the exposure.


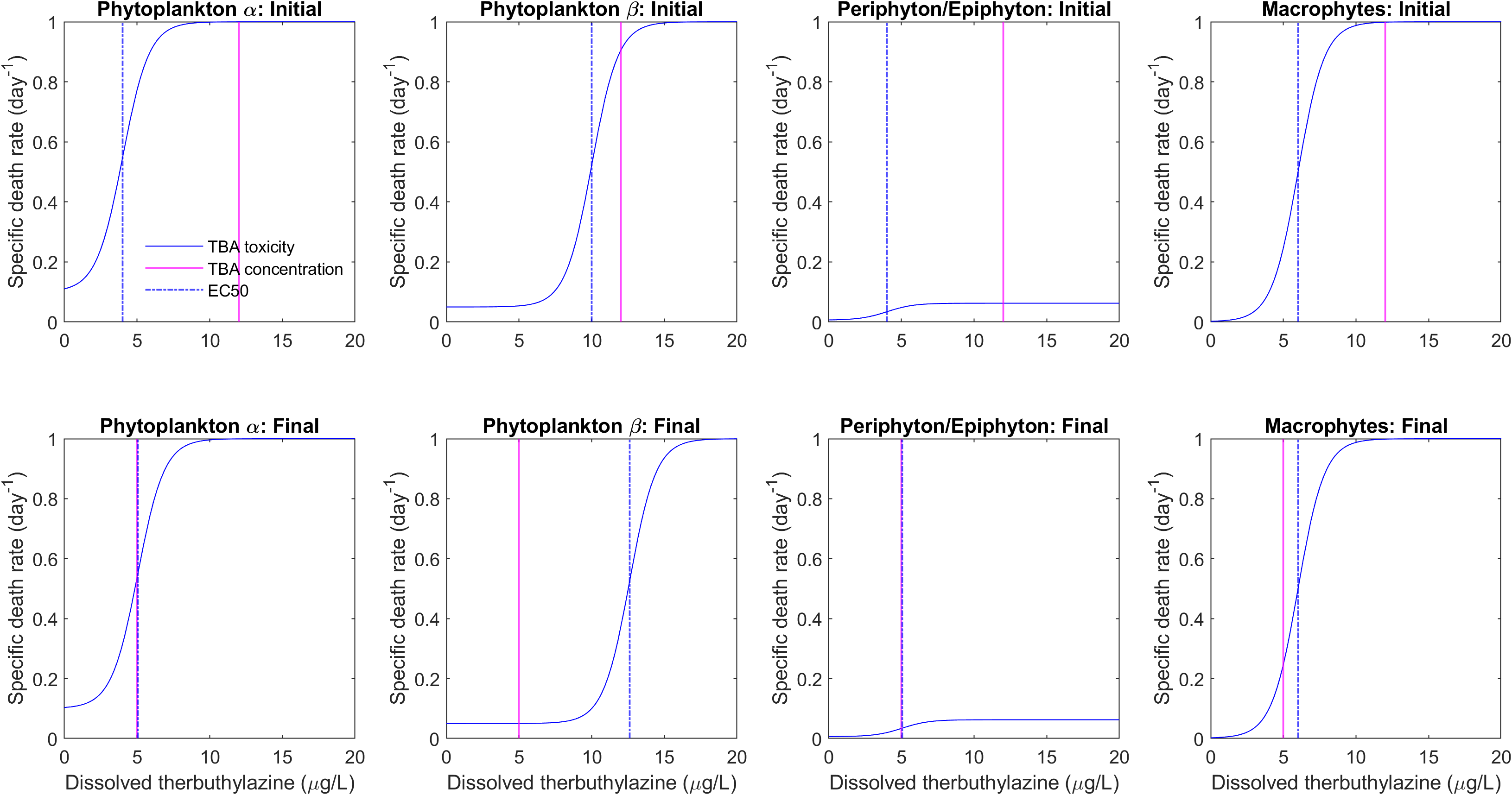


**Figure S2.** Timeline of the microcosm experiment, including preparation and final sampling.


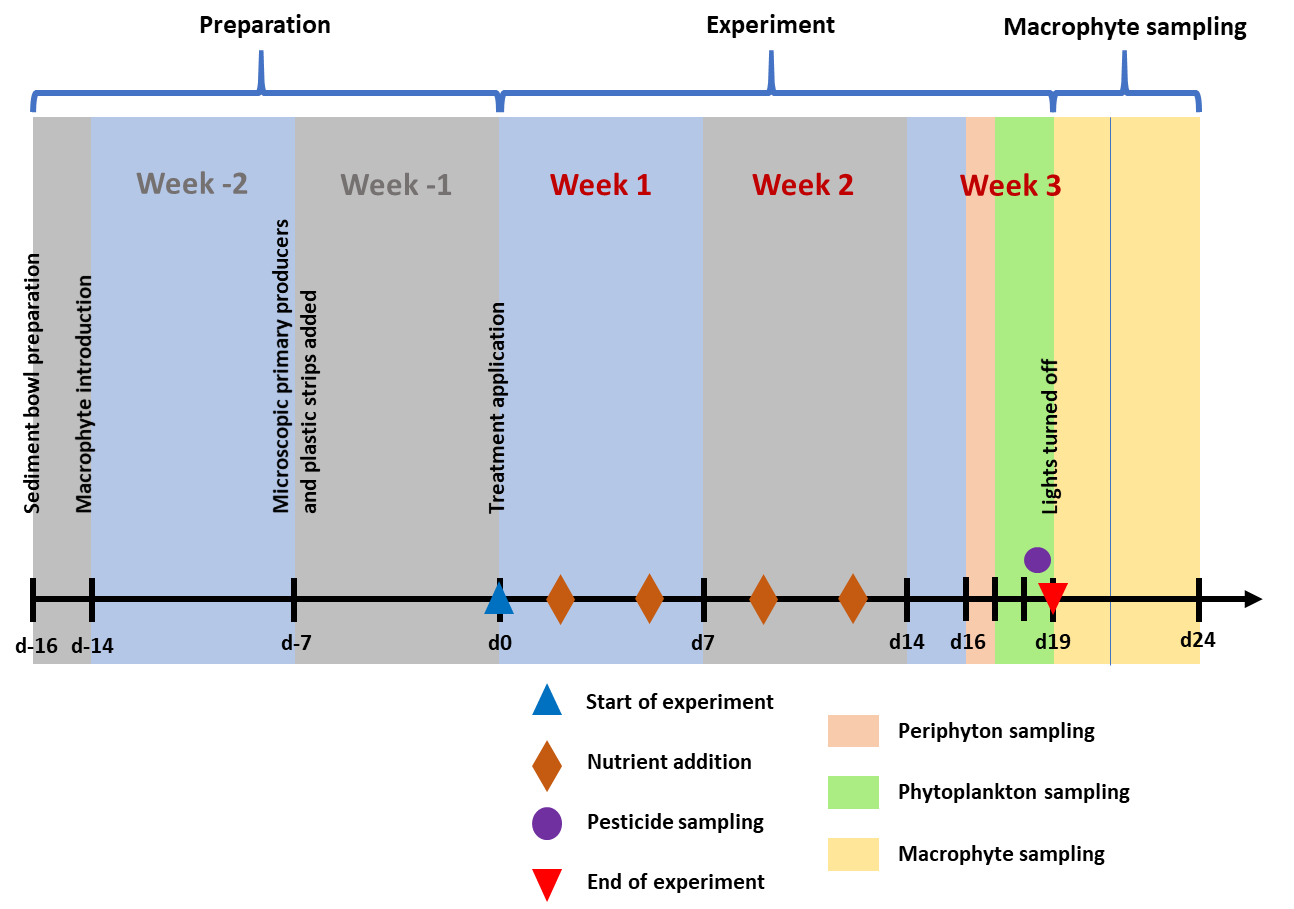


**Figure S3.** Temporal dynamics of state variables at the lower temperature (22°C) under scenario D2 in (a) the control (top panels); and (b) the highest ARO treatment (ARO 16x, bottom panels).

| **(a)** | 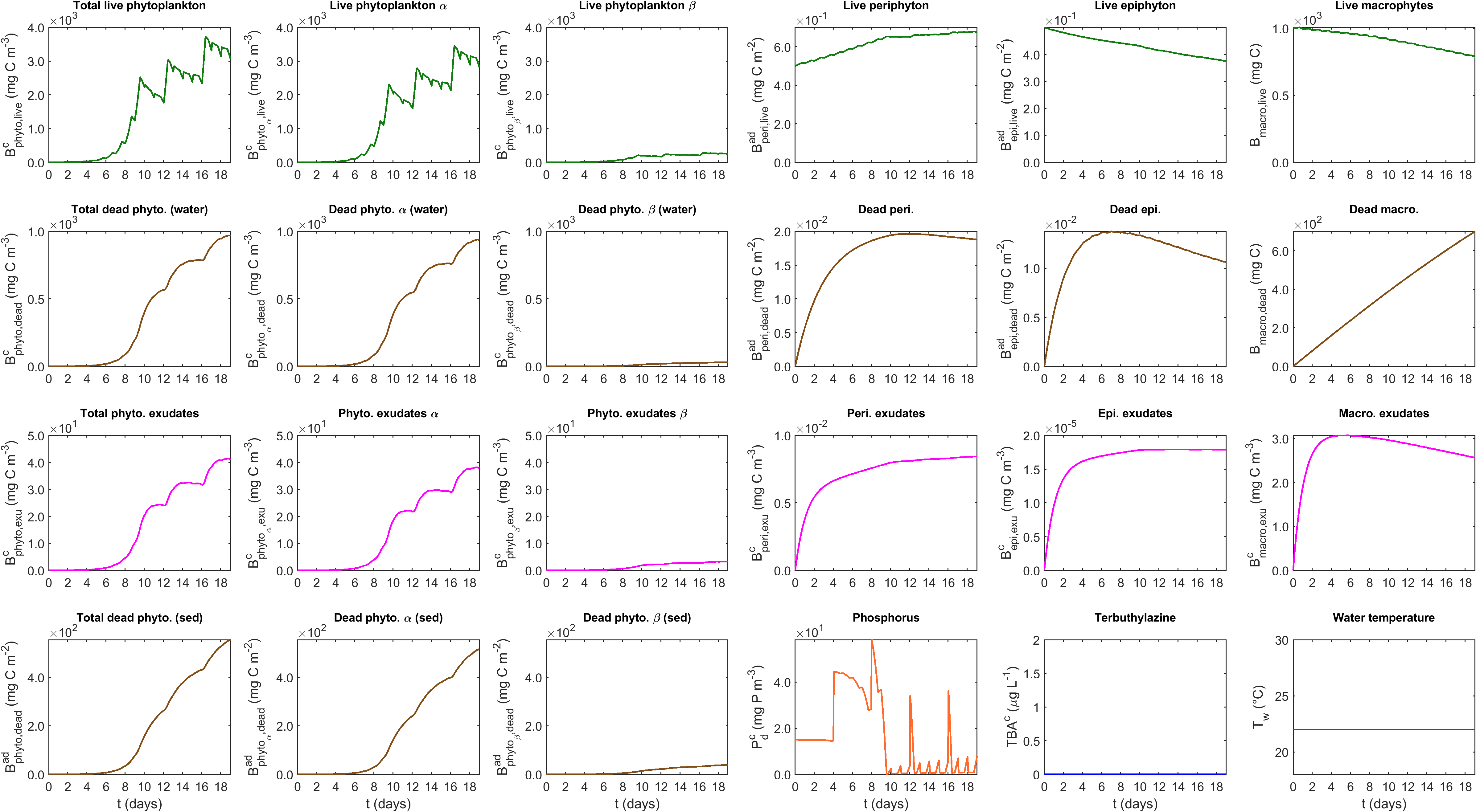 |
| --- | --- |
| **(b)** | 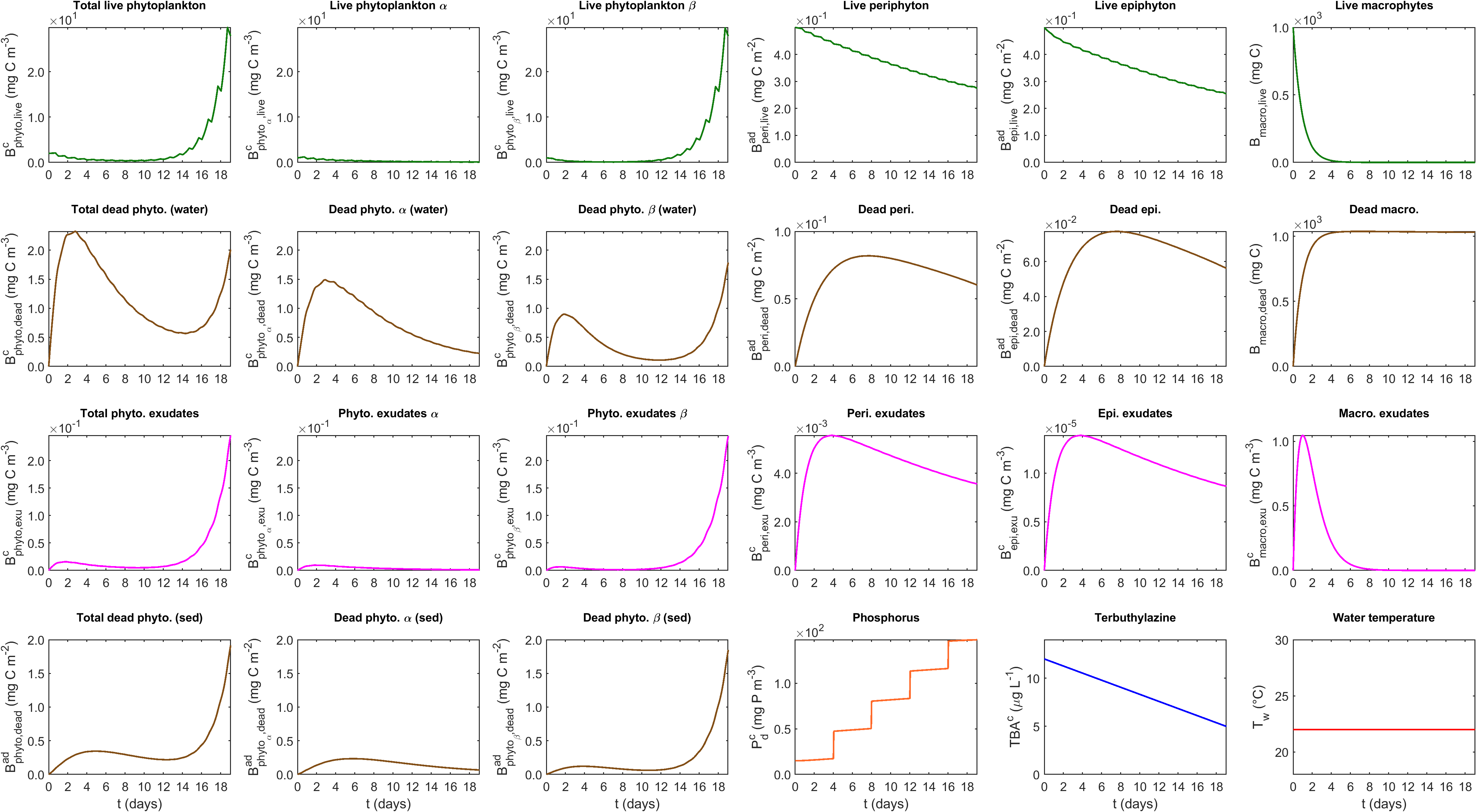 |

**Figure S4.** Temporal dynamics of state variables at the higher temperature (26°C) under scenario D2 in (a) the control (top panels); and (b) the highest ARO treatment (ARO 16x, bottom panels).

| **(a)** | 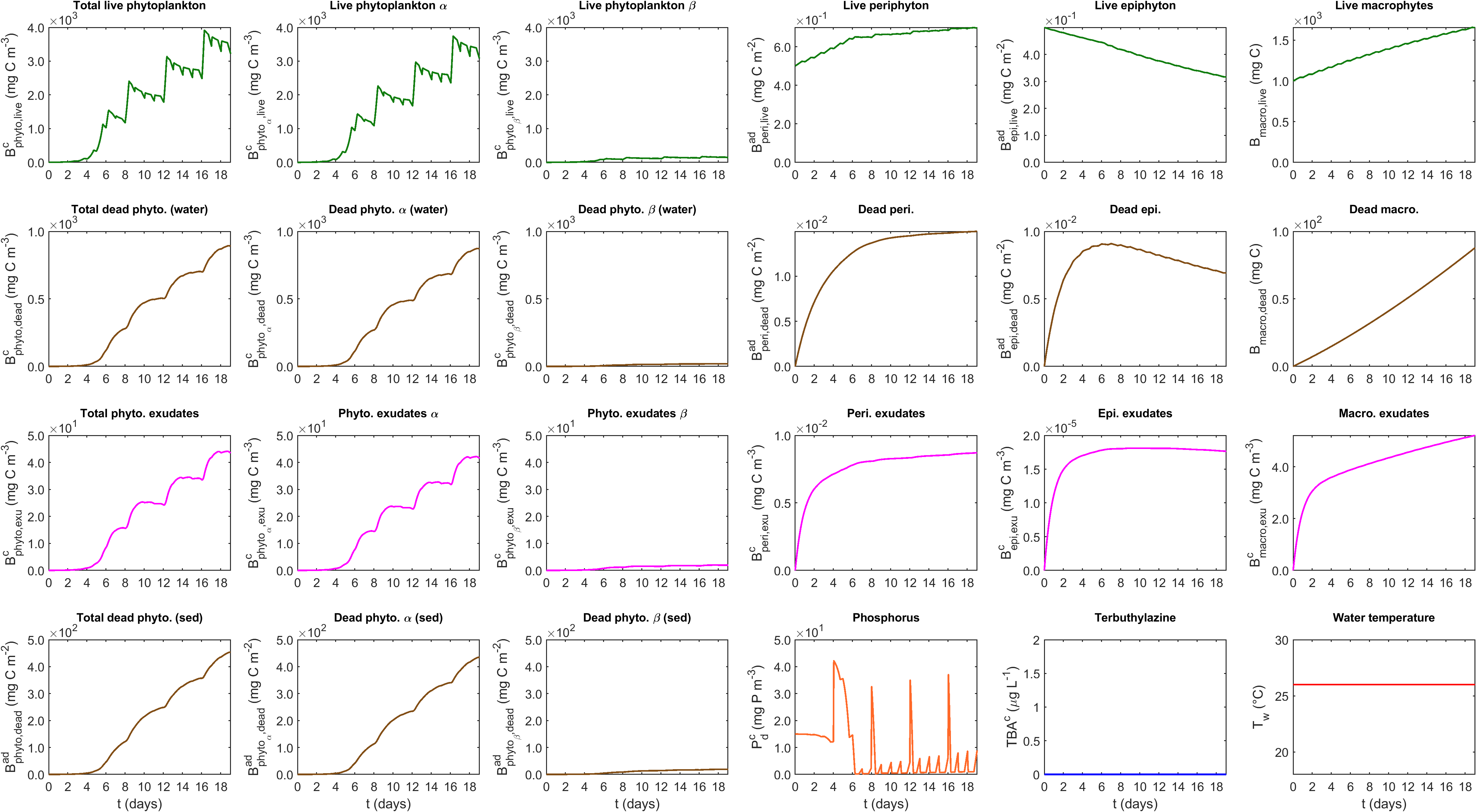 |
| --- | --- |
| **(b)** | 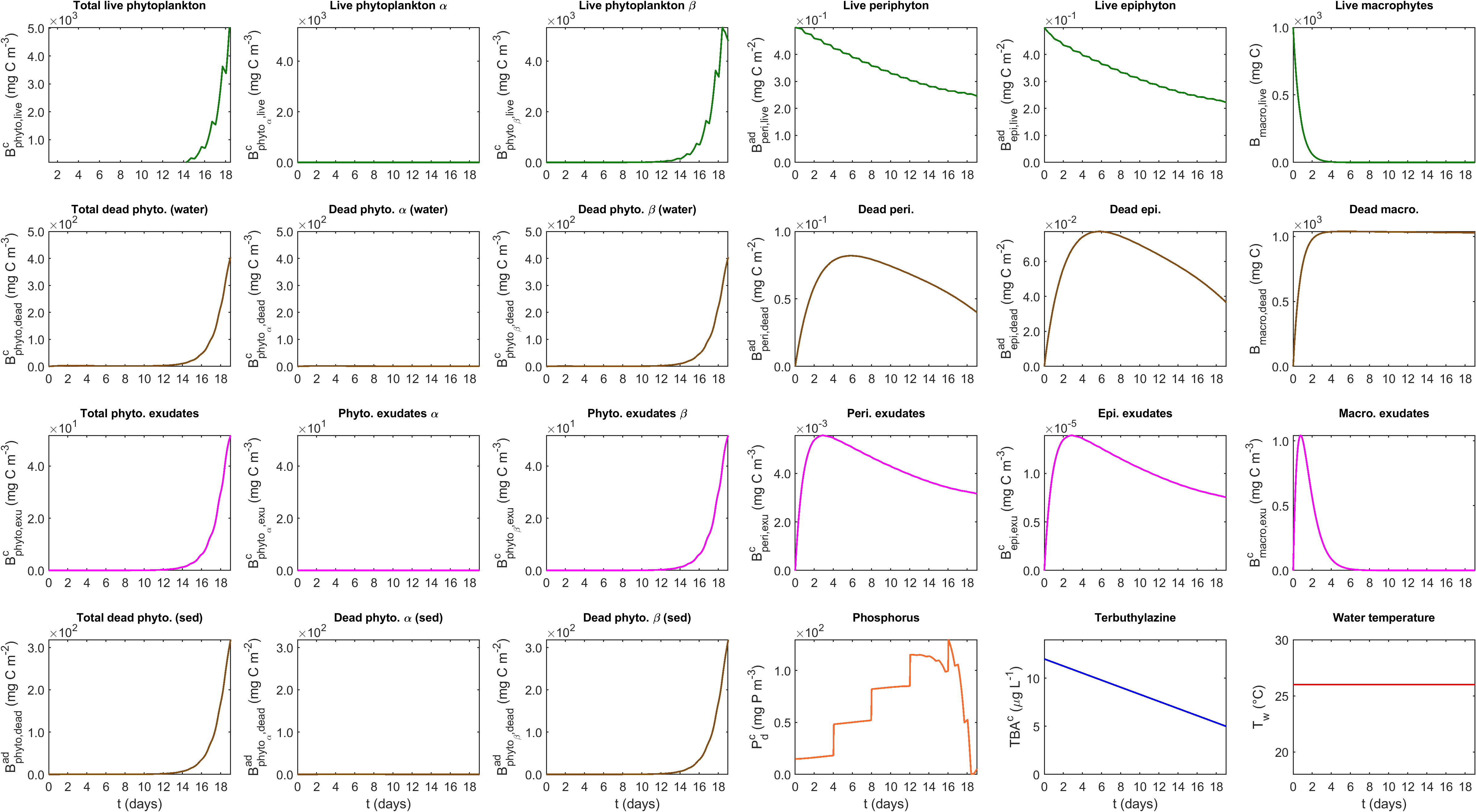 |

**Figure S5.** Temporal dynamics of the phosphorus, light and space limitation factors (PLF, LLF and SLF, respectively) for epiphyton under scenario D2 for the case of a mixed phytoplankton community (set 3) in the ambient (full lines) and warm (dashed lines) temperature treatments.

**
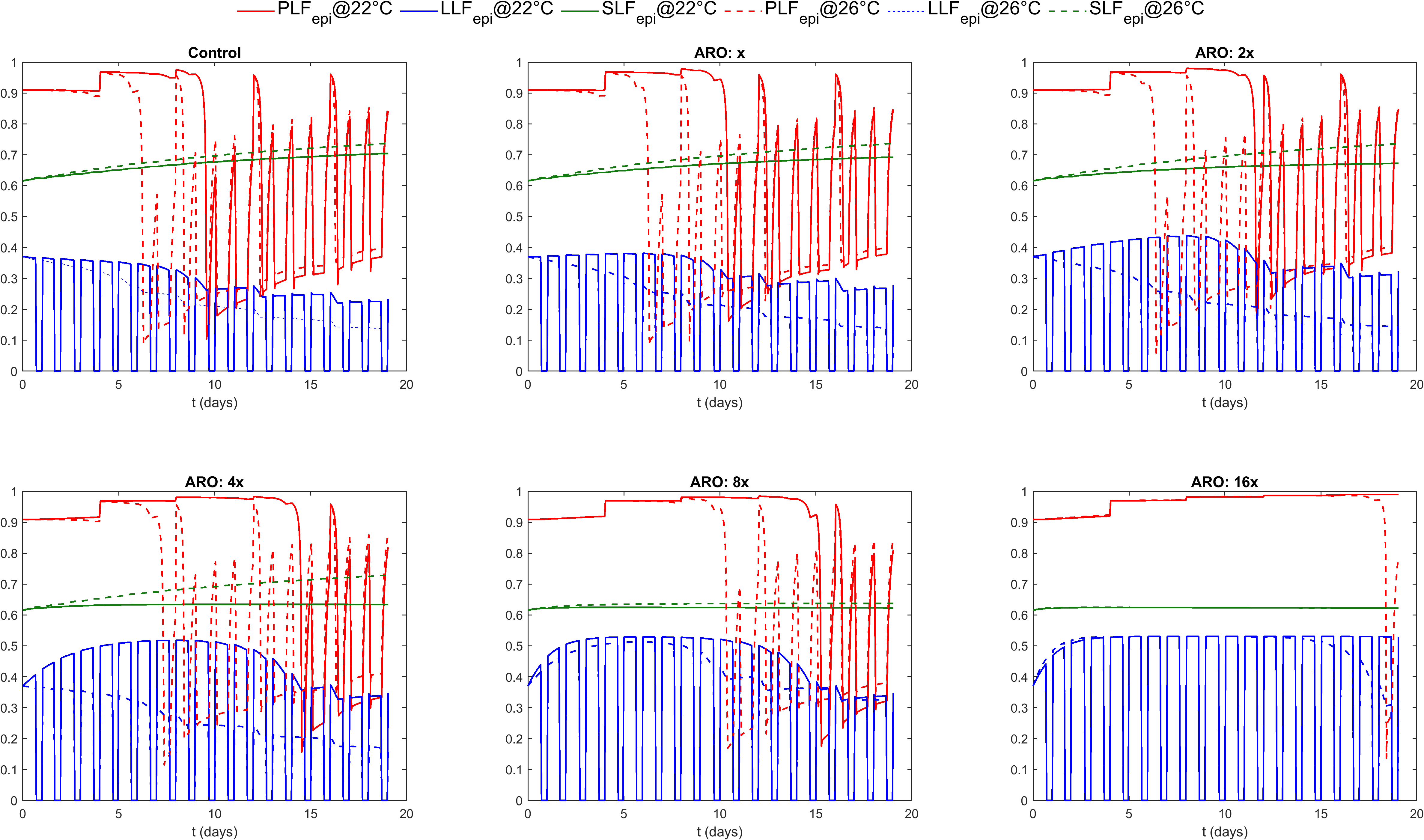
**

**Figure S6.** Example of mass balance controls implemented in the model for carbon and phosphorus, accounting for gains and losses over the course of each simulation.


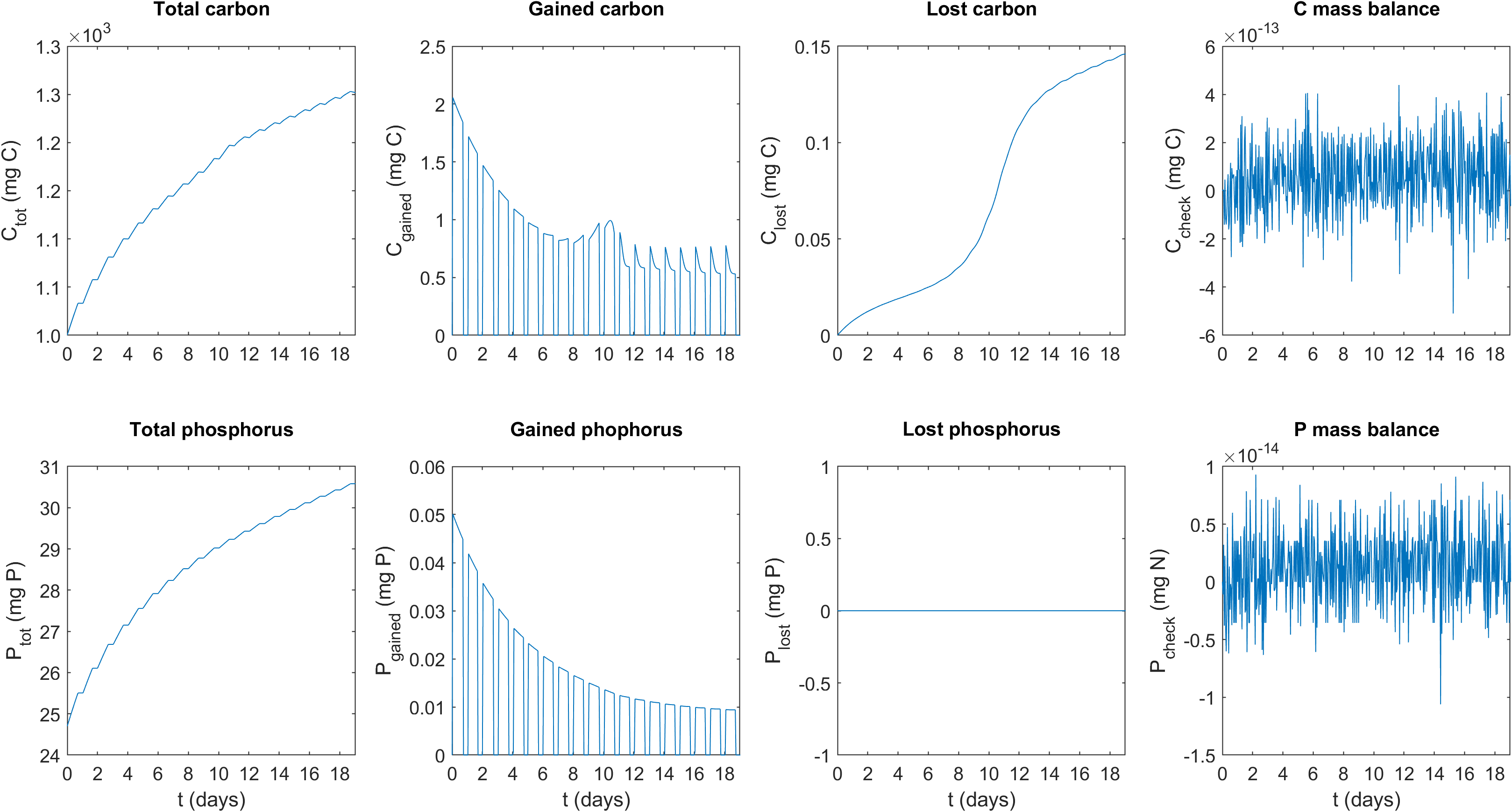


Table S1. Fixed parameters: descriptions, values, units of measurement and references/assumptions

| **Parameter** | **Description** | **Value** | **Units** | **References/assumptions** |
| --- | --- | --- | --- | --- |
| $k_{bg}$ | Background light extinction coefficient | 0.3 | m^-1^ | Value in the range of those used in other modeling studies (e.g., Jäger, et al., 2010; Peeters, et al., 2013) |
| $r_{S}$ | Fraction of incident shortwave irradiance that is reflected back from the water surface | 0.04 | (-) | Albedo of water is in the range of 0.02-0.05 except when the angle of incidence becomes nearly parallel to the surface |
| ${TBA}_{decay}$ | Rate of decay of terbuthylazine | 0.0307 | (86400s)^-1^ | On average, ~59% of the initial concentration of terbuthylazine could still be detected in the water samples taken 2 hours and 19 days after application of the treatments |
| $\theta_{j}$ | Pre-exponential factor, all processes  $j=p_{i},l_{d,i},l_{bg,i},b_{i,dead},b_{i,exu}$,  where  $i=phyto_{A},phyto_{B},peri,epi,macro$ | 1.07 | (-) | Typical value for all biological processes in several models (e.g., EPA WASP, Ambrose, et al., 1993) |
| $T_{w,ref,bio}$ | Reference water temperature for all biological and biochemical processes | 20 | °C | Reference value in most studies (e.g., Sand-Jensen & Borum, 1991; and Hilt et al. 2018) |

Table S2. Phytoplankton parameters: descriptions, values, units of measurement and references/assumptions

| **Parameter** | **Description** | **Value** | **Units** | **References/assumptions** |
| --- | --- | --- | --- | --- |
| $k_{live,spec,{phyto}_{g=\alpha,\beta}}$ | Specific light extinction coefficient, live phytoplankton biomass (both groups) | 3×10^-4^ | m^2^ mg C^-1^ | Jäger, et al., 2010; Peeters, et al., 2013 |
| $k_{dead,spec,{phyto}_{g=\alpha,\beta}}$ | Specific light extinction coefficient, dead phytoplankton biomass (both groups) | 3×10^-4^ | m^2^ mg C^-1^ | Assumed similar to that of live phytoplankton biomass |
| $\mu_{ref,{phyto}_{\alpha}}$ | Reference fractional growth rate of fast-growing phytoplankton (group *α*) | 1.5 | (86400s)^-1^ | In the range of other studies (e.g., Jäger, et al., 2010; Peeters, et al., 2013) |
| $\mu_{ref,{phyto}_{\beta}}$ | Reference fractional growth rate of slow-growing phytoplankton (group *β*) | 1.0 | (86400s)^-1^ | In the range of other studies (e.g., Jäger, et al., 2010; Peeters, et al., 2013) |
| $l_{d,ref,{phyto}_{\alpha}}$ | Reference fractional death rate of fast-growing phytoplankton (group *α*) | 0.10 | (86400s)^-1^ | Assumed rate for group A |
| $l_{d,ref,{phyto}_{\beta}}$ | Reference fractional death rate of slow-growing phytoplankton (group *β*) | 0.05 | (86400s)^-1^ | Reduced death rate for group B with respect to group A |
| $l_{exu,ref,{phyto}_{g=\alpha,\beta}}$ | Reference fractional exudation rate of phytoplankton biomass (both groups) | 0.01 | (86400s)^-1^ | Jäger, et al., 2010; Peeters, et al., 2013 |
| $w_{live,{phyto}_{g=\alpha,\beta}}$ | Sinking velocity of live phytoplankton biomass (both groups) | 0.05 | m (86400s)^-1^ | A very low sinking velocity was assumed because microcosms were subjected to artificial bubbling |
| $w_{dead,{phyto}_{g=\alpha,\beta}}$ | Sinking velocity of dead phytoplankton biomass (both groups) | 0.05 | m (86400s)^-1^ | A very low sinking velocity was assumed because microcosms were subjected to artificial bubbling |
| $b_{dead,ref,{phyto}_{g=\alpha,\beta}}$ | Reference fractional biodegradation rate of dead phytoplankton biomass (both groups) | 0.3 | (86400s)^-1^ | Assumed rate for the biodegradation of dead unicellular organisms |
| $b_{exu,ref,{phyto}_{g=\alpha,\beta}}$ | Reference fractional biodegradation rate of phytoplankton exudates (both groups) | 0.8 | (86400s)^-1^ | Assumed rate for the biodegradation of cellular exudates |
| $q_{P,{phyto}_{g=\alpha,\beta}}$ | Phosphorus-to-carbon cellular quota of phytoplankton cells (both groups) | 0.0244 | mg P mg C^-1^ | Mass-based Redfield ratio |
| $m_{P,{phyto}_{g=\alpha,\beta}}$ | Half-velocity in the Monod-type formulation of the phosphorus limitation factor for phytoplankton (both groups) | 1.5 | (86400s)^-1^ | Jäger, et al., 2010; Peeters, et al., 2013 |
| $I_{sw,opt,{phyto}_{g=\alpha,\beta}}$ | Optimum shortwave irradiance for phytoplankton (both groups) | 57.1 | W m^-2^ | Aalderink & Jovin, 1997 |

Table S3. Periphyton parameters: descriptions, values, units of measurement and references/assumptions

| **Parameter** | **Description** | **Value** | **Units** | **References/assumptions** |
| --- | --- | --- | --- | --- |
| $\mu_{ref,peri}$ | Reference fractional growth rate of periphyton | 1.5 | (86400s)^-1^ | Assumed similar to that of fast-growing phytoplankton (group A) |
| $l_{d,ref,peri}$ | Reference fractional death rate of periphyton | 0.1 | (86400s)^-1^ | Assumed similar to that of fast-growing phytoplankton (group A) |
| $l_{exu,ref,peri}$ | Reference fractional exudation rate of periphyton biomass | 0.01 | (86400s)^-1^ | Assumed similar to that of phytoplankton |
| $b_{dead,ref,peri}$ | Reference fractional biodegradation rate of dead periphyton cells | 0.3 | (86400s)^-1^ | Assumed rate for the biodegradation of dead unicellular organisms |
| $b_{exu,ref,peri}$ | Reference fractional biodegradation rate of periphyton exudates | 0.8 | (86400s)^-1^ | Assumed rate for the biodegradation of cellular exudates |
| $q_{P,peri}$ | Phosphorus-to-carbon cellular quota of periphyton cells | 0.0244 | mg P mg C^-1^ | Mass-based Redfield ratio |
| $m_{P,peri}$ | Half-velocity in the Monod-type formulation of the phosphorus limitation factor for periphyton | 1.5 | (86400s)^-1^ | Assumed similar to that for phytoplankton |
| $m_{Sp,peri}$ | Half-saturation constant for the space limitation factor for periphyton | 4×10^-6^ | m^2^ | Assumed value |
| $I_{sw,opt,peri}$ | Optimum shortwave irradiance for periphyton | 57.1 | W m^-2^ | Assumed similar to that for phytoplankton (from Aalderink & Jovin, 1997) |

Table S4. Epiphyton parameters: descriptions, values, units of measurement and references/assumptions

| **Parameter** | **Description** | **Value** | **Units** | **References/assumptions** |
| --- | --- | --- | --- | --- |
| $\mu_{ref,epi}$ | Reference fractional growth rate of epiphyton | 1.5 | (86400s)^-1^ | Assumed similar to that of fast-growing phytoplankton (group A) |
| $l_{d,ref,epi}$ | Reference fractional death rate of epiphyton | 0.1 | (86400s)^-1^ | Assumed similar to that of fast-growing phytoplankton (group A) |
| $l_{exu,ref,epi}$ | Reference fractional exudation rate of epiphyton biomass | 0.01 | (86400s)^-1^ | Assumed similar to that of phytoplankton |
| $b_{dead,ref,epi}$ | Reference fractional biodegradation rate of dead epiphyton cells | 0.3 | (86400s)^-1^ | Assumed rate for the biodegradation of dead unicellular organisms |
| $b_{exu,ref,epi}$ | Reference fractional biodegradation rate of epiphyton exudates | 0.8 | (86400s)^-1^ | Assumed rate for the biodegradation of cellular exudates |
| $q_{P,epi}$ | Phosphorus-to-carbon cellular quota of epiphyton cells | 0.0244 | mg P mg C^-1^ | Mass-based Redfield ratio |
| $m_{P,epi}$ | Half-velocity in the Monod-type formulation of the phosphorus limitation factor for epiphyton | 1.5 | (86400s)^-1^ | Assumed similar to that for phytoplankton |
| $m_{Sp,epi}$ | Half-saturation constant for the space limitation factor for epiphyton | 5×10^-4^ | m^2^ | Assumed value |
| $I_{sw,opt,epi}$ | Optimum shortwave irradiance for epiphyton | 57.1 | W m^-2^ | Assumed similar to that for phytoplankton (from Aalderink & Jovin, 1997) |
| ${shad}_{epi}$ | Fraction of incident light that is absorbed by epiphyton | 0.083 | (-) | Coefficient reported in Köhler et al., 2010 |

Table S5. Macrophyte parameters: descriptions, values, units of measurement and references/assumptions

| **Parameter** | **Description** | **Value** | **Units** | **References/assumptions** |
| --- | --- | --- | --- | --- |
| $k_{live,macro}$ | Specific light extinction coefficient of live macrophyte carbon biomass | 0.02 | m^2^ mg C^-1^ | Assumed value |
| $k_{dead,macro}$ | Specific light extinction coefficient, dead macrophyte carbon biomass | 0.02 | m^2^ mg C^-1^ | Assumed value |
| ${HB}_{ratio,macro}$ | Height:carbon biomass ratio for macrophytes | 5×10^-5^ | m mg C^-1^ | Assumed value |
| ${AB}_{ratio,macro}$ | Surface area:carbon biomass ratio for macrophytes | 8×10^-7^ | m^2^ mg C^-1^ | Assumed value |
| $\mu_{ref,macro}$ | Reference fractional growth rate for macrophytes | 0.20 | (86400s)^-1^ | Assumed value for the ensemble of three macrophyte species |
| $l_{d,ref,macro}$ | Reference fractional death rate for macrophytes | 0.001 | (86400s)^-1^ | Assumed value for the ensemble of three macrophyte species |
| $l_{exu,ref,macro}$ | Reference fractional exudation rate for macrophytes | 5×10^-5^ | (86400s)^-1^ | Assumed value for the ensemble of three macrophyte species |
| $b_{dead,ref,macro}$ | Reference fractional biodegradation rate of dead macrophyte biomass | 5×10^-4^ | (86400s)^-1^ | Assumed rate for the biodegradation of dead macrophyte biomass |
| $b_{exu,ref,macro}$ | Reference fractional biodegradation rate of macrophyte exudates | 0.8 | (86400s)^-1^ | Assumed rate for the biodegradation of cellular exudates |
| $q_{P,macro}$ | Phosphorus-to-carbon cellular quota of macrophytes | 0.0244 | mg P mg C^-1^ | Mass-based Redfield ratio |
| $h_{macro}$ | Half-saturation constant in the light limitation factor for macrophytes | 22 | W m^-2^ | Studies on *P. pectinatus* (e.g., Hootsmans & Vermaat, 1994). |
| $\sigma_{T,macro}$ | Temperature constant in the temperature mediation factor for macrophytes | 20 | °C | Hilt et al. 2018 |
| $T_{w,opt,macro}$ | Optimum water temperature for macrophytes | 20 | °C | Hilt et al. 2018. |

Table S6. Group- and scenario-specific toxicokinetic parameters values for each group of primary producers

|  |  | Scenario | | | | | | | |
| --- | --- | --- | --- | --- | --- | --- | --- | --- | --- |
|  |  | A1 | A2 | B1 | B2 | C1 | C2 | D1 | D2 |
| ${\boldsymbol{EC}\boldsymbol{50}}_{\boldsymbol{TBA,ini,i}}$ | | | | | | | | | |
| Phytoplankton *α* | 22°C | 5.858 | 5.858 | 5.858 | 4.000 | 2.000 | 2.000 | 2.000 | 2.000 |
|  | 26°C | 5.858 | 8.000 | 5.858 | 5.000 | 2.000 | 5.000 | 2.000 | 4.000 |
| Phytoplankton *β* | 22°C | 12.000 | 12.000 | 12.000 | 10.000 | 8.000 | 8.000 | 8.000 | 8.000 |
|  | 26°C | 12.000 | 15.000 | 12.000 | 11.000 | 8.000 | 11.000 | 8.000 | 10.000 |
| Periphyton | 22°C | 5.858 | 5.858 | 5.858 | 4.000 | 2.000 | 2.000 | 2.000 | 2.000 |
|  | 26°C | 5.858 | 8.000 | 5.858 | 5.000 | 2.000 | 5.000 | 2.000 | 4.000 |
| Epiphyton | 22°C | 5.858 | 5.858 | 5.858 | 4.000 | 2.000 | 2.000 | 2.000 | 2.000 |
|  | 26°C | 5.858 | 8.000 | 5.858 | 5.000 | 2.000 | 5.000 | 2.000 | 4.000 |
| Macrophytes | 22°C | 5.858 | 5.858 | 1.500 | 1.500 | 5.858 | 5.858 | 3.000 | 3.000 |
|  | 26°C | 5.858 | 8.000 | 1.500 | 2.500 | 5.858 | 8.000 | 3.000 | 6.000 |
| $\boldsymbol{f}_{\boldsymbol{TBA,i}}$ | | | | | | | | | |
| Phytoplankton *α* | 22°C | - | - | - | - | - | - | 4.000 | 4.000 |
|  | 26°C | - | - | - | - | - | - | 4.000 | 2.500 |
| Phytoplankton *β* | 22°C | - | - | - | - | - | - | 4.000 | 4.000 |
|  | 26°C | - | - | - | - | - | - | 4.000 | 2.500 |
| Periphyton | 22°C | - | - | - | - | - | - | 4.000 | 4.000 |
|  | 26°C | - | - | - | - | - | - | 4.000 | 2.500 |
| Epiphyton | 22°C | - | - | - | - | - | - | 4.000 | 4.000 |
|  | 26°C | - | - | - | - | - | - | 4.000 | 2.500 |
| Macrophytes | 22°C | - | - | - | - | - | - | 1.333 | 1.333 |
|  | 26°C | - | - | - | - | - | - | 1.333 | 1.167 |

Table S7. Initial values for all state variables

| **State variable** | **Description** | **Initial value** | **Units** |
| --- | --- | --- | --- |
| $C_{live,phyto,\alpha}$ | Volume concentration of live phytoplankton biomass (group *α*) | 1 (set 1)  0 (set 2)  1 (set 3) | mg C m^-3^ |
| $C_{live,phyto,\alpha}$ | Volume concentration of live phytoplankton biomass (group *β*) | 0 (set 1)  1 (set 2)  1 (set 3) | mg C m^-3^ |
| $C_{dead,phyto,\beta}$ | Volume concentration of dead phytoplankton biomass (group *α*) | 0 | mg C m^-3^ |
| $C_{dead,phyto,\beta}$ | Volume concentration of dead phytoplankton biomass (group *β*) | 0 | mg C m^-3^ |
| $C_{exu,phyto,\alpha}$ | Volume concentration of carbon in phytoplankton exudates (group *α*) | 0 | mg C m^-3^ |
| $C_{exu,phyto,\beta}$ | Volume concentration of carbon in phytoplankton exudates (group *β*) | 0 | mg C m^-3^ |
| $C_{live,peri}$ | Areal density of live periphyton biomass | 0.5 | mg C m^-2^ |
| $C_{dead,peri}$ | Areal density of dead periphyton biomass | 0 | mg C m^-2^ |
| $C_{exu,peri}$ | Volume concentration of carbon in periphyton exudates | 0 | mg C m^-3^ |
| $C_{live,epi}$ | Areal density of live epiphyton biomass | 0.5 | mg C m^-2^ |
| $C_{dead,epi}$ | Areal density of dead epiphyton biomass | 0 | mg C m^-2^ |
| $C_{exu,epi}$ | Volume concentration of carbon in epiphyton exudates | 0 | mg C m^-3^ |
| $C_{live,macro}$ | Live macrophyte biomass | 1000 | mg C |
| $C_{dead,macro}$ | Dead macrophyte biomass | 0 | mg C |
| $C_{exu,macro}$ | Volume concentration of carbon in macrophyte exudates | 0 | mg C m^-3^ |
| $C_{dead,sed,phyto,\alpha}$ | Areal density of dead phytoplankton biomass in the sediments (group *α*) | 0 | mg C m^-2^ |
| $C_{dead,sed,phyto,\beta}$ | Areal density of dead phytoplankton biomass in the sediments (group *β*) | 0 | mg C m^-2^ |
| $P_{d}$ | Volume concentration of dissolved inorganic phosphorus | 15 | mg P m^-3^ |

**Table S8. Nominal and measured ARO concentrations 2 hours & 18 days after its application (n=3, ARO 8: n=1, < d.l. = below detection limit). Concentrations in µg L^-1^.**

| Nominal | Terbuthylazine | Pirimicarb | Tebuconazole | Copper | NO_3_-N |
| --- | --- | --- | --- | --- | --- |
| 1 | 0.75 | 3.75 | 22.5 | 10.5 | 2250 |
| 2 | 1.5 | 7.5 | 45 | 21 | 4500 |
| 4 | 3 | 15 | 90 | 42 | 9000 |
| 8 | 6 | 30 | 180 | 84 | 18000 |
| 16 | 12 | 60 | 360 | 168 | 36000 |
|  |  |  |  |  |  |
| Day 0 | Terbuthylazine | Pirimicarb | Tebuconazole | Copper | NO_3_-N |
| 1 | 0.64 + 0.01 | 3.82 + 0.22 | 21.11 + 1.21 | n.a. | n.a. |
| 2 | 1.28 + 0.06 | 7.24 + 0.14 | 38.07 + 4.00 | n.a. | n.a. |
| 4 | 2.74 + 0.06 | 15.56 + 0.62 | 83.46 + 6.56 | n.a. | n.a. |
| 8 | 5.70 + 0.00 | 31.07 + 0.00 | 171.80 + 0.00 | n.a. | n.a. |
| 16 | 11.64 + 0.59 | 63.64 + 3.02 | 323.03 + 23.06 | n.a. | n.a. |
|  |  |  |  |  |  |
| Day 18 | Terbuthylazine | Pirimicarb | Tebuconazole | Copper | NO_3_-N |
| 1 | 0.44 ± 0.06 | 2.64 ± 0.75 | 9.62 ± 6.24 | n.a. | 0.00 ± 0.00 |
| 2 | 0.79 ± 0.08 | 4.94 ± 0.45 | 25.94 ± 2.60 | n.a. | 0.00 ± 0.00 |
| 4 | 1.77 ± 0.23 | 10.03 ± 1.53 | 54.32 ± 8.60 | n.a. | 0.29 ± 0.23 |
| 8 | 2.80 ± 0.84 | 16.61 ± 3.91 | 101.60 ± 21.23 | n.a. | 1.79 ± 0.25 |
| 16 | 9.07 ± 0.05 | 43.99 ± 3.64 | 264.14 ± 37.00 | n.a. | 4.38 ± 0.35 |

**References**

Aalderink, R. H., and Jovin, R. (1997). Estimation of the photosynthesis/irradiance (P/I) curve parameters from light and dark bottle experiments. *Journal of Plankton Research* 19 (11), 1713-1742.

Ambrose, Jr., R. B., Wool, T. A., and Martin, J. L. (1993). The Water Quality Analysis Simulation Program, WASP5 - Part A: Model Documentation. Athens(Georgia): Environmental Research Laboratory; AScI Corporation.

Hilt, S., Alirangues Nuñez, M. M., Bakker, E. S., Blindow, I., Davidson, T. A., Gillefalk, M., et al. (2018). Response of Submerged Macrophyte Communities to External and Internal Restoration Measures in North Temperate Shallow Lakes.” *Frontiers in Plant Science* 9 (194), 1-24.

Hootsmans, M. J. M., and Vermaat, J. E. (1994). Light-response curves of Potamogeton pectinatus L. as a function of plant age and irradiance level during growth. In: W. Van Vierssen, M. Hootsmans & J. Vermaat, eds. Lake Veluwe, a Macrophyte-dominated System under Eutrophication Stress (Geobotany, v. 21). Dordrecht: Springer Science+Business Media, pp. 62-117.

Hootsmans, M. J. M. (1994). A growth analysis model for Potamogeton pectinatus L. In: W. Van Vierssen, M. Hootsmans & J. Vermaat, eds. Lake Veluwe, a Macrophyte-dominated System under Eutrophication Stress (Geobotany, v. 21). Dordrecht: Springer Science+Business Media, pp. 250-286.

Jäger, C., Diehl, S., and Emans, M. (2010) Physical determinants of phytoplankton production, algal stoichiometry and vertical nutrient fluxes. *The American Naturalist* 175 (4): E91-E104.

Köhler, J., Hachoł, J., and Hilt, S. (2010). Regulation of submersed macrophyte biomass in a temperate lowland river: Interactions between shading by bank vegetation, epiphyton and water turbidity. *Aquatic Botany* 92 (2): 129-136.

Peeters, F., Kerimoglu, O. & Straile, D. (2013). Implications of seasonal mixing for phytoplankton production and bloom development. *Theoretical Ecology* 6, 115-129.

Redfield, A. C. (1958). The biological control of chemical factors in the environment. *American Scientist* 46 (3), 230A, 205-221.

Sager, J. C., and McFarlane, J. C. (1997). Radiation. In GROWTH CHAMBER HANDBOOK, NC-101 Regional Committee on Controlled Environment Technology and Use, Editor: R. W. Langhans und T. W. Tibbitts, 1-30.

Sand-Jensen, K., and Borum, J. (1991). Interactions among phytoplankton, periphyton, and macrophytes in temperate freshwaters and estuaries. *Aquatic Botany* 41 (1-3).

Sládeček, V., and Sládečková, A. (1964). Determination of the periphyton production by means of the glass slide method. *Hydrobiologia* 23 (1-2), 125-158.

Steele, J. H. (1962) Environmental control of photosynthesis in the sea. *Limnology and Oceanography* 7 (2), 137-150.

Wiley, P. E., Campbell, J. E., and McKuin, B. (2011). Production of Biodiesel and Biogas from Algae: A Review of Process Train Options. *Water Environment Research* 83 (4), 326-338.
